# Grapevine response to a *Dittrichia viscosa* extract and a *Bacillus velezensis* strain

**DOI:** 10.3389/fpls.2022.1075231

**Published:** 2022-12-16

**Authors:** Mélina Ramos, Núria Daranas, Mercè Llugany, Roser Tolrà, Emilio Montesinos, Esther Badosa

**Affiliations:** ^1^ Institute of Food and Agricultural Technology-CIDSAV-XaRTA, University of Girona, Girona, Spain; ^2^ Plant Physiology (BABVE), Faculty of Biosciences, Universitat Autònoma de Barcelona, Bellaterra, Spain

**Keywords:** biocontrol products, transcriptomics, RT-qPCR, gene markers, metabolites, grapevine

## Abstract

The present study aims to evaluate the response of the three Mediterranean local grapevines ‘Garnacha Blanca’, ‘Garnacha Tinta’, and ‘Macabeo’ to treatments with biocontrol products, namely a botanical extract (Akivi, *Dittrichia viscosa* extract) and a beneficial microorganism (*Bacillus* UdG, *Bacillus velezensis*). A combination of transcriptomics and metabolomics approaches were chosen in order to study grapevine gene expression and to identify gene marker candidates, as well as, to determine differentially concentrated grapevine metabolites in response to biocontrol product treatments. Grapevine plants were cultivated in greenhouse under controlled conditions and submitted to the treatments. Thereafter, leaves were sampled 24h after treatment to carry out the gene expression study by RT-qPCR for the three cultivars and by RNA-sequencing for ‘Garnacha Blanca’. Differentially expressed genes (DEGs) were investigated for both treatments and highly influenced DEGs were selected to be tested in the three cultivars as treatment gene markers. In addition, the extraction of leaf components was performed to quantify metabolites, such as phytohormones, organic acids, and phenols. Considering the upregulated and downregulated genes and the enhanced metabolites concentrations, the treatments had an effect on jasmonic acid, ethylene, and phenylpropanoids defense pathways. In addition, several DEG markers were identified presenting a stable overexpression after the treatments in the three grapevine cultivars. These gene markers could be used to monitor the activity of the products in field treatments. Further research will be necessary to confirm these primary results under field conditions.

## 1 Introduction

The European Union is the main world producer, consumer, and exporter of grapevine for wine-making (*Vitis vinifera*), and the production is mainly concentrated in three countries: Italy (29.7%), Spain (27.1%), and France (24.2%) (European Commission, 2021). Vineyards are threatened by several diseases, including powdery mildew and gray mold caused by the fungal pathogens *Erysiphe necator* and *Botrytis cinerea*, respectively, and downy mildew caused by the oomycete *Plasmopara viticola* (Avenard et al., 2003; Gessler et al., 2011; Reynier, 2011; Beris et al., 2021). These causal agents are able to infect several grapevine tissues starting from flowers and leaves (*E. necator*), from leaves (*P. viticola*), and from berries (*B. cinerea*). If the first infections are not controlled, the diseases spread quickly in the vineyard and mildews can infect berries as well. These diseases can cause severe crop losses depending on the season and the cultivation area, reducing the harvest quality and yield, plant vigor and photosynthesis (Calonnec et al., 2006; Leroy et al., 2013; Kunova et al., 2021).

The main grapevine cultivars are susceptible to these diseases and vineyard protection requires intensive treatments with plant protection products (PPPs), such as chemical fungicides from bud burst until ripening (Boubakri et al., 2013). The frequency average of the applied fungicide treatments is around ten treatments per year, which can rise up to 20 treatments under the most critical conditions (Butault et al., 2010; Leroy et al., 2013; Pertot et al., 2017). This intensive use of PPPs can affect the treated crops, the environment, and the consumer health as well (Alavanja et al., 2004; Boubakri et al., 2012; Boubakri et al., 2013; Krzyzaniak et al., 2018; Zambito Marsala et al., 2020). To prevent the negative impact of the intensive use of synthetic PPPs, more environmentally friendly compounds, such as biocontrol products are promoted by European governments (European Parliament, Council of the European Union, 2009a; European Parliament, Council of the European Union, 2009b). Among the different types of biocontrol products, there are natural substances derived from plant, animal, or mineral extracts and beneficial microorganisms able to protect the plant from pests and diseases.

Natural substances as well as beneficial microorganisms used as biocontrol products present modes of action mainly relying on (i) direct action against the pathogen (Bonaterra et al., 2012; Persaud et al., 2019) or (ii) indirect action by stimulating plant defense (Perazzolli et al., 2011; Perazzolli et al., 2012; Pieterse et al., 2014; Rienth et al., 2019; Nishad et al., 2020; Burdziej et al., 2021). It has been reported that a plant extract from *Vitis* can present direct activity in grapevine against downy mildew (Schnee et al., 2013). Some beneficial microorganisms are able to compete against pathogens for space and nutrient supplies (Bonaterra et al., 2012) or to show antagonism activity against pathogens through antimicrobial or lytic enzyme production (Ongena and Jacques, 2008; Wang et al., 2013; Mora et al., 2015; Vilà et al., 2016 ). Moreover, laminarin (algae extract) and chito-oligosaccharides associated with oligogalacturonides (COS-OGA) are already used in vineyards as plant defense stimulators, protecting grapevine against downy mildew and powdery mildew (Van Aubel et al., 2014; Bodin et al., 2020). Some beneficial microorganisms are already authorized and used in vineyards (Otoguro and Suzuki, 2018). It is reported *Bacillus subtilis* strains that show antagonism activity against gray mold (Maachia et al., 2015) and a *Saccharomyces cerevisiae* cell wall derivatives-based product that induce resistance against downy mildew, gray mold and powdery mildew (De Miccolis Angelini et al., 2019). Biocontrol products with a combination between the two types of mechanisms are described as well (Krzyzaniak et al., 2018; Esmaeel et al., 2020; Zhou et al., 2020).

Plant defense response to biotic stresses relies on different levels of recognition. After pathogen infection, molecular patterns or effectors of the pathogen are recognized leading to (pathogen-associated molecular patterns) PAMP-triggered immunity (PTI) or effector-triggered immunity (ETI). Both PTI and ETI stimulate plant systemic acquired resistance (SAR) (Abdul Malik et al., 2020). Beneficial microorganisms recognition can also trigger plant defense response called induced systemic resistance (ISR). SAR and ISR responses involve phytohormones, such as salicylic acid (SA), jasmonic acid (JA), and ethylene (ET), being SA more specific to SAR and JA/ET pathway to ISR (Pieterse et al., 2014). It is reported that SA is involved in the defense against biotrophic pathogens, including *P. viticola* and *E. necator*, whereas JA/ET pathway against necrotrophic pathogens, such as *B. cinerea.* However, the two pathways can be activated simultaneously (Burdziej et al., 2021). Direct application of phytohormones or analogues are able to trigger defense response in grapevine against downy mildew (Bodin et al., 2020; Burdziej et al., 2021). Despite biocontrol products modes of action are not always well-understood in several plant species and cultivars, it is important to assess that they have no impact on the treated plants or the environment.

This study aims to evaluate the response of three appreciated and autochthonous grapevine cultivars of the Mediterranean zone, concretely of Catalonia (Spain): Garnacha Blanca, Garnacha Tinta, and Macabeo to biocontrol product treatments. Two biocontrol products from different origins were investigated: a botanical extract (Akivi, *Dittrichia viscosa* extract) and a beneficial bacterial strain (*Bacillus* UdG, *Bacillus velezensis* living bacteria). Both products are still in development and the combination of a whole genome transcriptomics approach and a targeted metabolomics approach was chosen to elucidate the grapevine response to the treatments. The objectives of this work are: (i) to study grapevine gene expression response after biocontrol product treatment using transcriptomics; (ii) to identify robust gene marker candidates presenting stable differential expression after treatment within the three grapevine cultivars; and (iii) to determine grapevine metabolites variations after biocontrol product treatment using targeted metabolomics.

## 2 Materials and methods

### 2.1 *Bacillus* UdG production and plant extract

*Bacillus velezensis* UdG strain was isolated from a wild plant collected during a sample screening as reported by Mora et al. (2011). *B. velezensis* UdG was routinely cultivated on a Luria-Bertani agar and incubated at 28°C for 24h. For the assays, two different products consisting of lyophilized and fresh cells were prepared.

For lyophilized *Bacillus* (BL), a fermentation process was done in a pilot-scale bioreactor (Biostat^®^ C, Sartorius, Germany) with a working volume of 30 L of production medium for 48 h at 28°C, pH7 and agitation ramp from 50 to 500 rpm. The production medium consisted of a modification of the original recipe of Walker and Abraham (1970). Specifically, the following modifications were considered: 7 g L^-1^ instead of 1 g L^-1^ of KH_2_PO_4_, 1 g L^-1^ instead of 4 g L^-1^ of L-monosodium glutamate, 5 g L^-1^ of molasses and 1 g L^-1^ of soy flour instead of 342 g L^-1^ of saccharose, 1 mL L^-1^ instead of 5 mL L^-1^ of ferric citrate solution, and 1 mL L^-1^ of oligoelement solution at 0.1 mg mL^-1^ instead of at 0.1 mg L^-1^. After fermentation, the cells were harvested by centrifugation (SA-1-02-175, GEA Westfalia, Granollers, Spain) at 10,000 rpm and the concentrated cell suspension was mixed with skimmed milk (15% final concentration). The bacterial suspension was frozen at −70°C and lyophilized in a laboratory scale freeze-dryer (Unitop HL, VirTis, Gardiner, NY). Dried samples were stored in vacuum sealed plastic-coated aluminum bags.

For fresh *Bacillus* (BF), a fermentation process was carried out in a 2-L Erlenmeyer flask for 48 h at 28°C and shaking at 150 rpm with 500 mL of the original recipe of production medium (modification: oligoelement solution was used at 0.1 mg mL^-1^ instead of at 0.1 mg L^-1^). After fermentation, the cells were harvested by centrifugation at 13,200 g for 10 min (Centrifuge 5810R, Eppendorf) and concentrated 10X with the corresponding volume of supernatant.

The plant extract Akivi was provided by S.A.S. AkiNaO (France). It is a formulated botanical extract prototype from *Dittrichia viscosa* composed of a high content of polyphenols and terpenes (Tamm et al., 2017).

### 2.2 Plant material, treatments, and experimental design

Three grapevine cultivars (*Vitis vinifera* L.), namely Garnacha Blanca, Garnacha Tinta and Macabeo, grafted on rootstock 110R, were obtained from commercial nurseries (Agromillora Iberica and Viveros Villanueva Vides, Spain). One-year-old bench-grafted grapevine rootlings were planted in a 2 L pot with 80% of the growing media (Prodeasa BV35, Burés Profesional, Spain), 20% of perlite (A-13, Agroteibe, Spain), and 4 g of the fertilizer (Osmocote^®^ Exact Mini 3-4M, ICL Specialty Fertilizers, France). Bench-grafted grapevines were grown in a greenhouse at 25 ± 2°C, 60 ± 10% relative humidity and a 16:8 h light:dark photoperiod. Young stocks with at least about 4 to 6 expanded leaves were used for the experiments.

The treatments consisted of Akivi at 0.521 g L^-1^ (Aki), and *Bacillus* UdG at 10^8^ CFU mL^-1^ lyophilized (BL) and fresh (BF). Water was used as the solvent to prepare each treatment. The BF treatment was only used in the experiment with cv. Garnacha Blanca. A non-treated control (NTC) using water was included in all the experiments. The products were sprayed on adaxial and abaxial leaf surfaces using an airbrush until near run-off.

The experimental design for cv. Garnacha Blanca stocks included 4 randomized blocks corresponding to the different treatment modalities (Aki, BL, BF, and NTC), while for cvs. Garnacha Tinta and Macabeo included 3 blocks (Aki, BL, and NTC). Each block was composed of 4 biological replicates of 5 plants.

### 2.3 Sampling plant material and RNA isolation

Sampling was carried out 24 h after spraying plants with the products. At that time transcriptomics response, as well as, phytormone signalization and metabolomics responses can be evaluated. Four biological replicates were sampled for each treatment for RNA-sequencing analysis (RNA-seq), and three biological replicates for reverse transcription quantitative PCR (RT-qPCR) analysis. Two leaves from each plant (5 plants per biological replicate) were harvested, grounded, and soaked in liquid nitrogen. Each ground leaf sample was added to 2 mL tubes containing two borosilicate glass beads in order to obtain a fine powder using Tissuelyzer II system (Qiagen, USA) for 1 min at 30 Hz.

For total RNA isolation from grapevine leaves, the commercial kit Spectrum™ Plant Total RNA Kit (Sigma-Aldrich, USA) was used (**Supplementary Table 1**) following manufacturer’s instructions. Residual DNA was removed using Invitrogen™ TURBO DNA-free™ Kit (Applied Biosystems, USA).

The concentration and purity of RNA was assessed by spectrophotometric measurements using NanoDrop ND-1000 Spectrophotometer (Thermo Fisher Scientific, USA). RNA quality was evaluated using electrophoresis on 1.0% agarose gels.

Prior to RNA-seq analysis, a R.I.N. measurement was carried out using an Agilent 2100 Bioanalyzer (Agilent technologies, USA) to check RNA integrity from cv. Garnacha Blanca samples and RNA extracted in each sample was quantified by using the Qubit 2.0 Fluorometer (Invitrogen, USA).

### 2.4 RNA-sequencing and reads mapping

The plant response to treatments using transcriptomics was studied on cv. Garnacha Blanca grapevine leaves after spray application with Aki, BL, BF or water (NTC). A total of 16 samples were used for the library construction.

The RNA-seq transcriptome library was prepared using the TruSeq Stranded mRNA Sample Prep kit (Illumina, USA) following the manufacturer’s instructions using 1-2 µg of good quality RNA (R.I.N. > 7) as input. The RNA was fragmented by 3 minutes at 94°C and each purification step was performed by using 0.81X Agencourt AMPure XP beads. Final libraries were quantified by using the Qubit 2.0 Fluorometer (Invitrogen, USA) and quality tested by Agilent 2100 Bioanalyzer RNA Nano assay (Agilent technologies, USA). Libraries were then processed with Illumina cBot for cluster generation on the flowcell, following the manufacturer’s instructions and sequenced on paired-end (2x150 bp, 30M reads per sample) at the multiplexing level requested on NovaSeq6000 (Illumina). The CASAVA 1.8.2 version of the Illumina pipeline was used to process raw data for both format conversion and de-multiplexing.

Raw sequence files were first subjected to quality control analysis by using FastQC v0.10.1 (https://www.bioinformatics.babraham.ac.uk/projects/fastqc/) before trimming and removal of adapters with BBDuk (https://jgi.doe.gov/data-and-tools/bbtools/) setting a minimum base quality of 25 and a minimum read length of 35 bp. Reads were then mapped against the *V. vinifera* L. genome (*V. vinifera* cv. Pinot noir var. PN40024) (version 12X Ensembl) with STAR v2.6 (https://www.ncbi.nlm.nih.gov/pmc/articles/PMC3530905/). FeatureCounts v1.6.1 (https://academic.oup.com/bioinformatics/article/30/7/923/232889) was then used to obtain raw expression counts for each annotated gene using only uniquely mapping reads (MAPQ>=30). The differential gene expression (DGE) analysis was conducted with the R package edgeR (https://www.ncbi.nlm.nih.gov/pmc/articles/PMC2796818/) using the Trimmed mean of M-values (TMM) normalization method and considering as significant the genes with a False Discovery Rate (FDR) ≤ 0.05. Fragments Per Kilobase Million (FPKM) were obtained with edgeR. Gene Ontology (GO) Enrichment Analysis was performed using in-house scripts based on the AgriGO publication (https://academic.oup.com/nar/article/45/W1/W122/3796337). GO enrichment analysis was carried out using a threshold value (p-Value) < 0.05. The main biological functions were selected considering the Gene Ontology (GO) terms that showed at least 4 affected DEGs. Then the selected GO terms were analyzed using REVIGO web platform (http://revigo.irb.hr/) in order to summarize GO terms by removing redundancies. For each biological function category, different GO terms clusters (representative groups) that presented semantic similarity were obtained. The affected DEGs corresponding to all the GO terms of each cluster were added. In addition, GO terms that presented a background number over 1000 genes (BG-Item) were discarded since they are general GO terms. Clusters that showed less than 10 DEGs were joined under the term “other” considering the total number of genes. In addition, metabolic pathways influenced by the treatments were defined using Kyoto Encyclopedia of Genes and Genomes (KEGG) annotation (Kanehisa et al., 2016). KEGG pathways with a corrected p-Value < 0.05 were considered significantly influenced by the treatments.

### 2.5 Screening of differentially expressed genes

Screening of DEGs was carried out for each treatment modality (Aki, BL and BF) in comparison with the NTC. The two *Bacillus* modalities were studied together in order to identify common genes exclusively due to the bacterial activity, eliminating the effect of the freeze-drying.

Gene expression levels were assessed on the basis of unique mapped genes and were calculated using the FPKM method. FPKM values were used to analyze the differences in gene expression between treatments (Aki, BL, and BF) and NTC, by calculating a Fold-Change (FC) value.

Due to the high biological variability, the DEGs screening was conducted on the three biological replicates that presented less variability between each other, in order to avoid hiding a part of the treatment impact on the plant. DEGs exclusively altered by each treatment were targeted. The criteria of selection during the screening were based on DEGs presenting high differential expression value, specifically Log_2_(FC) > |1.4| and good repeatability among the three biological replicates.

### 2.6 Validation of DEGs by RT-qPCR

To confirm the transcriptome data obtained by RNA-seq analysis, 27 DEGs were selected (Log_2_(FC) > |1.4| and good repeatability among the three biological replicates) and their expression level was validated by RT-qPCR (**Supplementary Table 2**). The UBQ gene, coding for the Ubiquitin-conjugating enzyme, was used in this study as the endogenous gene for data normalization. This endogenous gene was previously selected according to the method described by Silver et al. (2006) (**Supplementary Figure 1**).

Standard curves for DEGs and the endogenous gene were obtained using decimal dilutions of extracted recombinant plasmid DNA (target sequences were cloned into a vector pSpark^®^ in *Escherichia coli* DH5α cells) corresponding to copy numbers ranging between 10^2^ and 10^7^. Ct values in each dilution were measured in triplicate and a negative non-template control was included in each run. Real-time PCR reactions included 10 μL SYBR^®^ Green PCR Master Mix (Applied Biosystems), 6 μL RNase-free water, 1 μL of each forward and reverse primer (**Supplementary Table 2**) at the corresponding concentration, and 2 μL DNA in a final volume of 20 μL. The optimal primer concentration (100, 300 or 600 nM) was previously defined. The thermal cycling conditions were as follows: 10 min at 95˚C for initial denaturation; 40 cycles of 15 s at 95˚C, and 1 min at 60˚C; and a final melting curve program of 60 to 95˚C with a heating rate of 0.5˚C s^-1^. Ct values were plotted against the logarithm of their initial template copy numbers and each standard curve was generated by a linear regression of the plotted points. The efficiency of each standard curve was calculated using the formula E = (10^(-1/a)^ -1) x100, where “a” is the slope of the curve.

For RT-qPCR, total RNA was extracted from leaf samples of treated plants using Spectrum™ Plant Total RNA Kit (Sigma-Aldrich) as explained above. First-strand of cDNA was synthetized from RNA using the High-Capacity cDNA Reverse Transcription Kit (Applied Biosystems) according to the manufacturer’s instructions. The absence of chromosomal DNA contamination was confirmed by minus-reverse transcriptase control in qPCR. Quantitative PCR was carried out in a QuantStudio™ 5 Real-Time PCR System (Applied Biosystems) to assess the transcriptional level of 27 DEGs. All the information of the selected genes and primers designed by Primer-BLAST tool from the Nacional Centre for Biotechnology Information (NCBI) are shown in **Supplementary Table 2**. Optimized qPCR reactions and the thermal cycling conditions were described above. Each qPCR assay included duplicates of each cDNA sample, no-template and RNA controls to check for contamination. Ct values from three biological replicates were averaged, and UBQ gene was used for data normalization.

The comparative critical threshold (ΔΔCt) method was used to assess the relative quantification of gene expression. Similar amplification efficiencies of all gene primer pairs were checked (**Supplementary Table 3**) making the ΔΔCt method appropriate to calculate the Fold-Change (FC). The ΔCt of the NTC leaf samples was used as the calibrating condition to calculate the FC. Genes were considered to be up- or downregulated if their FC were at least two-fold (FC = 2^1^ or 2^−1^) higher or less than the calibrator condition (Livak and Schmittgen, 2001) and showed statistically significant differences with the NTC.

### 2.7 Metabolite analysis

Metabolite extractions were carried out from powdered samples of grapevine leaves of cvs. Garnacha Blanca, Garnacha Tinta, and Macabeo obtained 24 h after spraying them with Aki, BL, or water (NTC) as explained above.

For phytohormone extraction, 250 mg of fresh grapevine leaves were grounded in an ice-cold mortar with 750 μL of extraction solution (methanol:isopropanol:acetic acid; 20:79:1 by vol.) (Llugany et al., 2013). Then, the supernatant was collected after centrifugation at 1000 g for 5 min at 4°C. These steps were repeated two more times and pooled supernatants were lyophilized. Finally, samples were dissolved in 250 μL pure methanol and filtered with a Spin-X centrifuge tube filter of 0.22 μm cellulose acetate (Costar, Corning Incorporated, USA). Phytohormone quantification was done using a standard addition calibration curve spiking control plant samples with the standard solutions of gibberellin A1 (GA1), gibberellin A4 (GA4), methyl jasmonate (MeJA), salicylic acid (SA), (±)-jasmonic acid (JA), (+)-cis, trans-abscisic acid (ABA) and 1-aminocyclopropane-1-carboxylic acid (ACC) ranging from 5 to 250 ppb and extracting as described above. Deuterated hormones jasmonic acid-d5 (JA-d5) and salicylic acid-d6 (SA-d6) at 30 ppb and 300 ppb, respectively, were used as internal standards in all the samples and standards measurements. Standards were purchased from Merk (Germany).

Plant hormones were analyzed by LC-ESI-MS/MS system in multiple reaction monitoring mode (MRM) according to Segarra et al. (2006). Phytohormones were separated using HPLC Acquity (Waters, USA) on a Luna Omega C18 column 1.6 µm 100 Å 50 x 2.1 mm (Phenomenex, USA) at 50°C at a constant flow rate of 0.8 mL min^-1^ and 10 µl injected volume. The elution gradient was carried out with a binary solvent system consisting of 0,1% of formic acid in methanol (solvent A) and 0,1% formic acid in milli-Q H_2_O (solvent B) with the following proportions (v/v) of solvent A (t (min), %A): (0, 2) (0.2, 2), (1.6, 100), (2, 100), (2.1, 2) and (3, 2). MS/MS experiments were performed on an ABI 4000 Qtrap mass spectrometer (Sciex). All the analyses were performed using the Turbo Ionspray source in negative ion mode except for MeJA and ACC.

Quantification was made by injection of extracted and spiked samples in multiple reaction monitoring (MRM) mode. Identification of phytohormones was based on retention time and presence of peak in the MRM trace compared with those of the standards.

Organic acids (OA) were extracted with a classical extraction protocol. Briefly, 250 mg of fresh grapevine leave powder was grounded in an ice-cold mortar with 2 mL of hydrochloric acid (0.025N). Then, the supernatant was collected after centrifugation at 1000 g for 15 min at 4°C. Meanwhile, Sep-Pack C18 cartridges (Waters, USA) were activated with (i) 1.4 mL of methanol, (ii) 0.7 mL of milli-Q water, and (iii) 1.4 mL of hydrochloric acid (0,025M). Supernatant (1.4 mL) were passed through the cartridge to recover 0.7 mL of clean extract. Finally, samples were filtered at 0.22 μm just prior to injection into an HPLC system.

Organic acids were analyzed by HPLC-UV system (Shimazu, Japan) in the following conditions (Tolrà et al., 2005): YMC-Pack ODS-A HPLC column 5µm 120Å 250 x 4.6 mm (YMC, Germany) at a constant flow rate of 0.8 mL min^-1^ and 10 µl injected volume. The injection method ran 15 min with an isocratic flow of 50 nM Potassium dihydrogen phosphate (KH_2_PO_4_) adjusted at a pH of 2.8 using Phosphoric acid (H_3_PO_4_).

The following standards were used for OA measurements: acetic acid, cis-aconitic acid, trans-aconitic acid, ascorbic acid, citric acid, isocitric acid, formic acid, fumaric acid, galacturonic acid, gluconic acid, glucuronic acid, glutamic acid, glycine, glycolic acid, glyoxylic acid, lactic acid, maleic acid, malic acid, malonic acid, oxalic acid, oxoglutaric acid, pyruvic acid, quinic acid, succinic acid, tannic acid, and tartaric acid.

Four peaks corresponding to OA were detected on samples HPLC-UV chromatograms. Then, these peaks’ retention times were compared with the retention times of 26 standards injected in the same conditions, and the identification was confirmed by standard enrichment injection within the grapevine samples. The four OA were identified (oxalic acid, tartaric acid, malic acid, and oxoglutaric acid) and quantified thanks to calibration curves. Calibration curves: malic acid (y=1.2967x+7.0154, R^2 =^ 0.9967), oxalic acid (y=0.2891x+4.7116, R^2 =^ 0.9993), oxoglutaric acid (y=1.4261x+17.324, R^2 =^ 0.9972), tartaric acid (y=2.6801x+2.4512, R^2 =^ 0.9998).

Putative identification was carried out by comparing the retention time of the standards with the peaks obtained in the grapevine leaf samples. The standard addition to the samples was done to check that the standard matches the targeted peak in leaf matrix conditions. Calibration curves were done at an appropriate range for each putatively identified organic acid and R² must be above >0.99 to allow quantification. Quantification was made within the samples using the calibration curves.

Phenolic compounds were extracted according to Solecka et al. (1999) with modifications (Kidd et al., 2001). Briefly, leaves were extracted with 70% methanol and after centrifugation (10 min, 5000 x g) the supernatant was re-extracted three times with ethyl ether to eliminate ether soluble lipids. The remaining water phase was treated with 2 M HCl for acid hydrolysis of soluble conjugated phenolic compounds. After extraction with ethyl acetate and drying, the residue was re-dissolved in 50% methanol. Total phenolic compounds levels were determined by spectrophotometry (Shimadzu UV-2450, Duisburg, Germany) following the method of Folin-Ciocalteau (Singleton et al., 1999), using gallic acid (Sigma, Steinheim, Germany) as the standard with detection at 765 nm. The results were expressed in Gallic Acid Equivalents (GAE).

### 2.8 Statistical analysis

Principal Component Analysis (PCA) was applied to the RNA-seq data comparing the biological replicates of each treatment modality with the NTC. The statistical analysis of the RT-qPCR data was done using REST2009 Software (Pfaffl et al., 2002). DEGs standard curves for gene expression quantification were made by linear regression on Excel. Validation of the DEGs was performed by a correlation study between the gene expression measured by RNA-seq and RT-qPCR techniques. Pearson correlation analysis was applied to the data for each treatment modality using R software (R version 3.5.2).

For metabolite measurements, to identify significant differences between treated (Aki and BL) and NTC leaves, several statistical tests were performed. All tests were performed on R software (R version 3.5.2) with a significant level of p-Value < 0.05. First, each of the metabolite datasets were tested (Shapiro-Wilk and Bartlett tests) to determine the suitability of parametric or non-parametric tests. For parametric tests, one-way analysis of variance (ANOVA) was carried out followed by a Tukey’s multiple comparisons test. For non-parametric tests, Kruskal-Wallis test followed by Dunn test were carried out.

## 3 Results

### 3.1 Quality assessment of RNA-seq data and gene expression estimation

The 16 sequencing samples produced around 39 million of total sequencing raw reads for NTC and Aki treatments, while around 36 million for BL and BF treatments (**Supplementary Table 4**). Following the filtering and trimming process, around 33 (NTC and Aki) and 31 (BL and BF) millions of cleaned reads were obtained (85 and 86%, respectively, of the total sequencing reads).

When the reads were paired and aligned to the reference *V. vinifera* L. PN40024 genome, around 14.7 (BL and BF) and 15.8 (NTC and Aki) million reads from each treatment could be mapped, (94.9 and 94.6%, respectively, of the input paired reads) (**Supplementary Table 5**). Moreover, between 85.6 (NTC and Aki) and 86.7% (BL and BF) of the input paired reads were assigned to genes.

The overall quality of the experiment was evaluated considering the consistency between the biological replicates using the normalized gene expression values (normalization of the FPKM) from each treatment. The PCA analysis revealed that one out of four biological replicates of each treatment (Aki_R1, BL_R1, BF_R1, NTC_R3) did not cluster as expected from the experimental design (**Supplementary Figure 2**). This variability among replicates could hide some of the treatment effect on gene expression, thus, this replicate was not included in further analysis.

PCA on normalized gene expression using the three retained biological replicates showed that the two first principal components explained 83.57% (Aki), 84.21% (BL) and 85.43% (BF) of the variance. In addition, the PC1 explained 63.33% (Aki), 60.5% (BL) and 49.38% (BF) of the variability in gene expression between each treatment and the NTC.

The RNA-seq raw transcriptomic data were submitted to the GEO repository of the National Center for Biotechnology Information (NCBI) (GSE211268).

### 3.2 Analysis of the differential expression of genes after the treatments

Gene transcription in cv. Garnacha Blanca grapevine leaves was triggered by Aki, BL and BF treatments to varying degrees. The volcano plots show the degree of variation of the Differential Expression of Genes (DEGs) based on red and green dots (**Supplementary Figure 3**). The relationship between the fold-change (Log_2_(FC)) and the statistical significance of the differential expression test (-Log_10_(FDR)) is displayed.

Plot similarities within *Bacillus* treatments (BL and BF) were observed since the most of genes were distributed between Log_2_(FC) values of -4 and 4 and with significance values (-Log_10_(FDR)) up to 75 (downregulated genes) and 50 (upregulated genes). However, Akivi plot differed from *Bacillus* ones since the main of genes were distributed between Log_2_(FC) values of -3 and 3 and with lower significance values of 20 (downregulated genes) and 60 (upregulated genes).

Additionally, heatmaps of these DEGs for each treatment effect are shown in **Supplementary Figure 4**. The expression patterns of DEGs were consistent within the three biological replicates but differed between treatments in comparison with the NTC. After Aki and BL treatments, the number of genes that were over-expressed (red) and down-expressed (green) in comparison with the NTC were equivalent. However, after BF treatment a higher number of genes were over-expressed (red) in comparison with the NTC.

**Figure 1:**
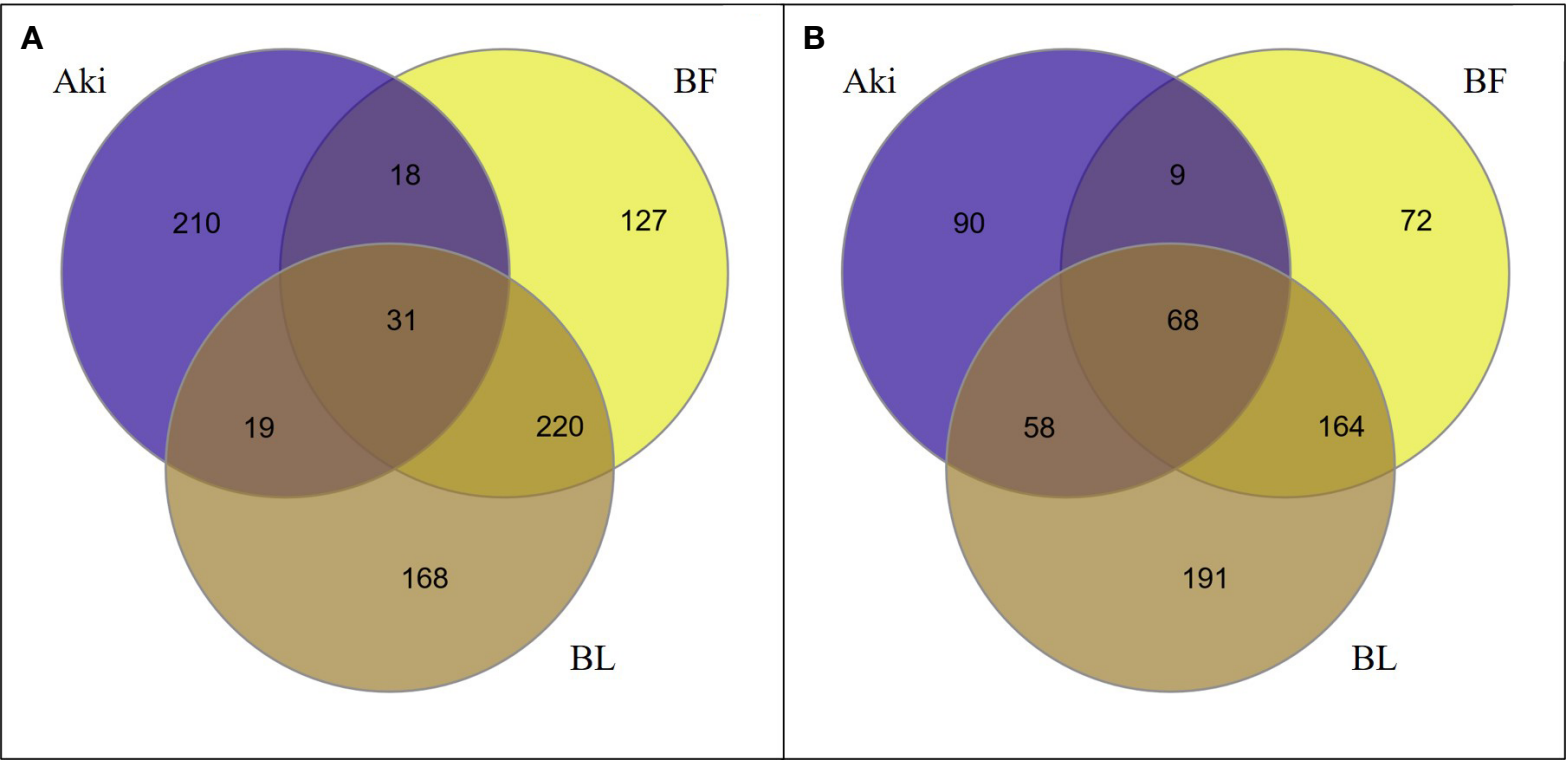
Venn diagrams showing the relationship between upregulated **(A)** and downregulated **(B)** differentially expressed genes (DEGs) identified in leaves of cv. Garnacha Blanca grapevine. Data correspond to 24h after treatments with Akivi (Aki), lyophilized (BL) and fresh (BF) *Bacillus* UdG, compared to the non-treated control (NTC).

As shown in Venn diagrams (**Figure 1**), 793 genes were upregulated and 652 genes were downregulated (log2(FC)>|1.4|) within the different treatments (Aki, BL and BF) in grapevine leaves after the treatments. *Bacillus* treatments (BL and BF) altered the expression level of a higher number of genes than the botanical extract Akivi treatment (Aki). BL and BF treatments showed 438 and 396 upregulated DEGs, respectively, and 481 and 313 downregulated DEGs, respectively, whereas Aki treatment showed a total of 278 upregulated and 225 downregulated DEGs. In addition, the plant response towards *Bacillus* (both BL and BF) and Akivi (Aki) treatments was fairly specific since only 31 upregulated and 68 downregulated genes were common to all three treatments.

However, the *Bacillus* treatments, both lyophilized and fresh, shared a high number of up- (43.1%) and downregulated (41.3%) genes. These genes were altered by the *Bacillus* treatments, independently of being the product lyophilized or not. Therefore, these shared genes were used for the following validation of RNA-seq results by RT-qPCR. From the 583 upregulated genes after either BL or BF treatments, 251 genes were shared. From the rest of genes, 187 and 145 were only upregulated after BL and BF treatments, respectively. Whereas from the 562 downregulated genes after BL or BF treatments, 232 genes were shared. From the remaining genes, 249 and 81 were only upregulated after BL and BF, respectively.

### 3.3 Functional analysis of DEGs in grapevine after treatments

#### 3.3.1 GO analysis of DEGs

GO enrichment analysis was carried out to evaluate the major biological functions of DEGs influenced by the Aki, BL, and BF treatments. The biological functions are classified into three categories: biological process (BP), cellular component (CC), and molecular function (MF). Upregulated GO terms were identified in 34.4, 25.8 and 25.0% of DEGs after the Aki, BL and BF treatments, respectively, whereas 35.3, 32.9 and 41.4% were downregulated (**Supplementary Table 6**).

**Figure 2:**
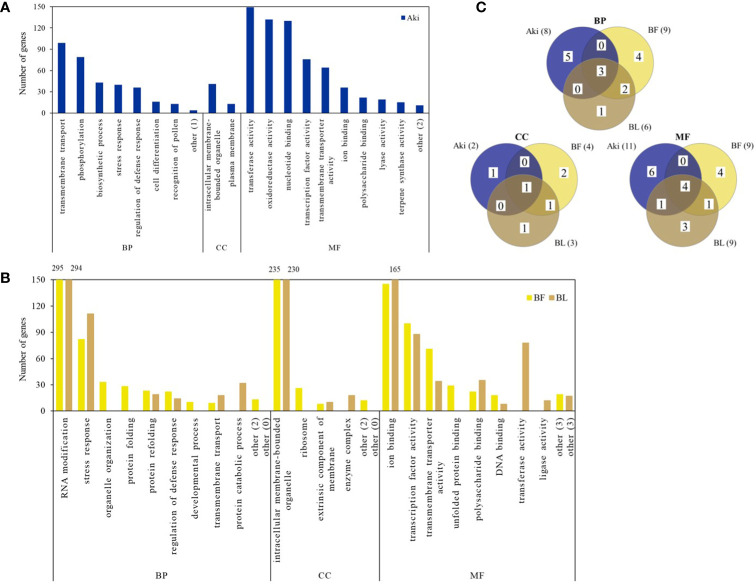
Upregulated genes according to Gene Ontology (GO) enrichment and REVIGO analysis in cv. Garnacha Blanca grapevine after treatment with Akivi (Aki), lyophilized (BL) and fresh (BF) *Bacillus* UdG, compared to the non-treated control (NTC). **(A, B)** Bar graphs show the number of upregulated DEGs in each GO term cluster. Clusters that showed less than 10 DEGs were included under the term “other”, indicating in parenthesis the number of clusters that represent. **(C)** Venn diagrams show the total upregulated GO term clusters. Categories of processes: biological (BP), cellular component (CC), and molecular function (MF).

**Figure 3:**
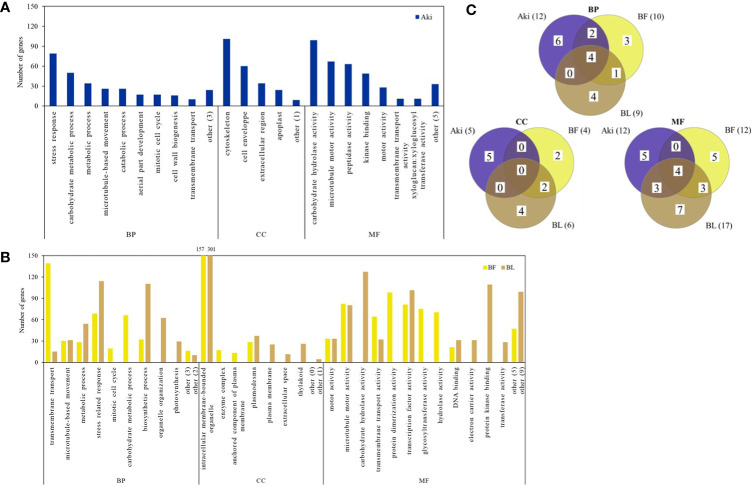
Downregulated genes according to Gene Ontology (GO) enrichment and REVIGO analysis in cv. Garnacha Blanca grapevine after treatment with Akivi (Aki), lyophilized (BL) and fresh (BF) *Bacillus* UdG, compared to the non-treated control (NTC). **(A, B)** Bar graphs show the number of downregulated genes in each GO term cluster. Clusters that showed less than 10 DEGs (BP and CC for Aki, BF and BL and MF for Aki) or 20 DEGs (MF for BF and BL) were included under the term “other”, indicating in parenthesis the number of clusters that represent. **(C)** Venn diagrams show the total downregulated GO term clusters. Categories of processes: biological (BP), cellular component (CC), and molecular function (MF).

**Figures 2**, **3** show the upregulated and downregulated GO term clusters obtained by REVIGO analysis. Three biological processes associated with upregulated genes, namely “transmembrane transport”, “stress response”, and “regulation of defense response” were shared by the three treatments. The GO term clusters “Phosphorylation”, “biosynthetic process”, “cell differentiation”, and “recognition of pollen” were exclusively enriched by Aki treatment, whereas “protein catabolic process”, “organelle organization”, “protein folding”, and “developmental process”, “RNA modification”, and “protein refolding” were related to by BL and/or BF treatments.

Four biological processes associated with downregulated genes, namely “metabolic process”, “microtubule-based movement”, “stress response” and “transmembrane transport” were shared by the three treatments. “Catabolic process”, “aerial part development”, and “cell wall biogenesis” were exclusively reduced by Aki treatment. Whereas “carbohydrate metabolic process” and “mitotic cell cycle” were reduced by both Aki and BF treatments, “organelle organization”, “photosynthesis”, and “biosynthetic process” were related to BF and/or BL treatments.

**Figure 4:**
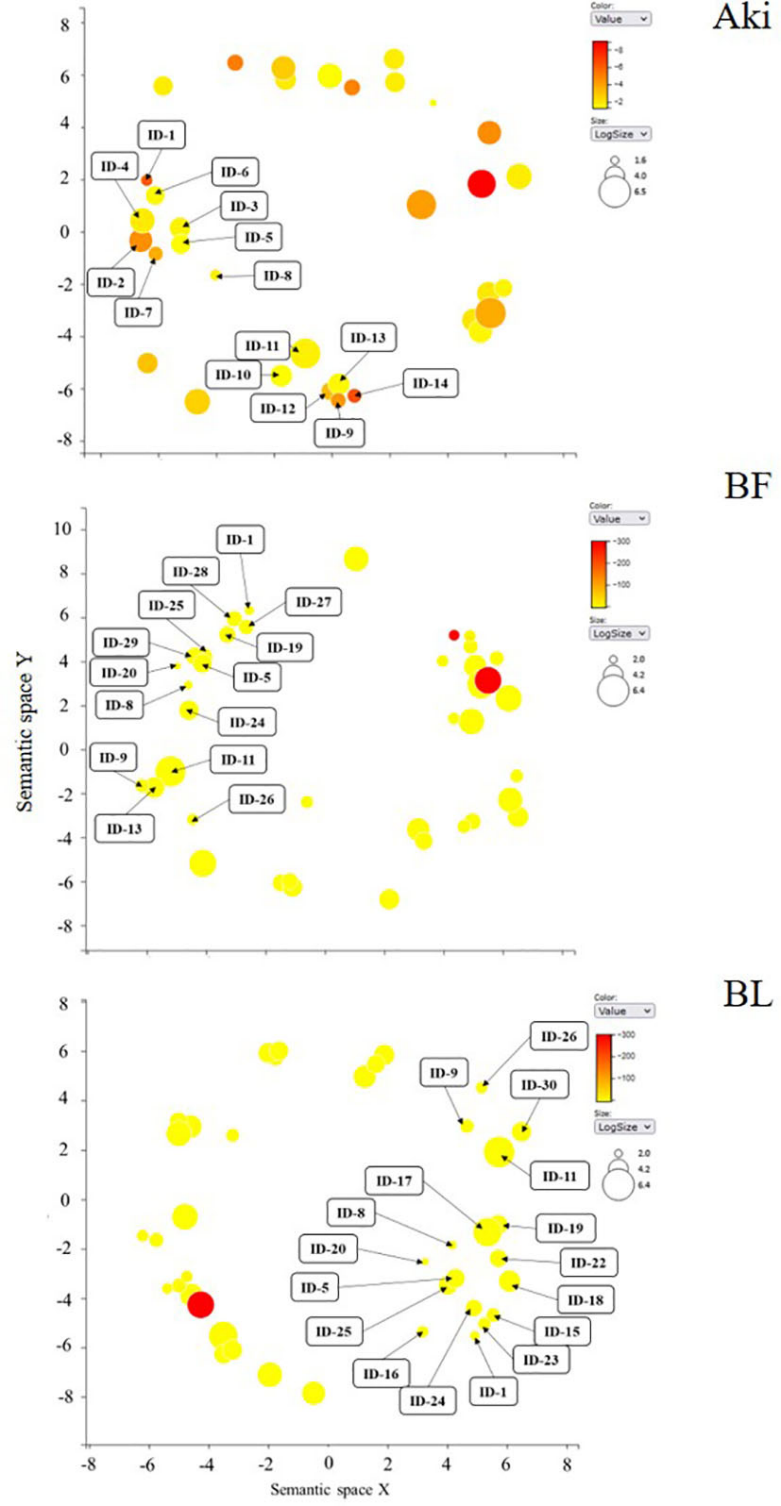
REVIGO graphs of upregulated GO term clusters (regulation of defense response and stress response) in leaves of cv. Garnacha Blanca grapevine included in biological process category after treatments with Akivi (Aki), lyophilized (BL) and fresh (BF) *Bacillus* UdG. ID: identification of GO terms associated with **Table 1**.

Some of the upregulated GO terms from BP category that were arranged in two well-defined clusters are related to plant defense response, namely “stress response” and “regulation of defense response” (**Figure 4**). These two clusters include 30 GO terms (**Table 1**). In general, only 6 out of 30 GO terms were shared by Aki and *Bacillus* (BF and/or BL) treatments. Five GO terms were shared by the two *Bacillus* treatments (BF and BL), while eight, three, and eight GO terms were unique for Aki, BF and BL, respectively.

**Table 1:** Representative groups (clusters) of upregulated GO terms of biological processes obtained with REVIGO and associated to plant defense responses, after treatments of cv. Garnacha Blanca grapevine leaves with Akivi (Aki), lyophilized (BL) and fresh (BF) *Bacillus* UdG.

Representative group	ID	GO ID: GO Term	Uniqueness*		
			Aki	BF	BL
Regulation of defense response	9	GO:2000022: regulation of jasmonic acid mediated signalling pathway			
	13	GO:0031347: regulation of defense response			
	26	GO:0051096: positive regulation of helicase activity			
	14	GO:0010112: regulation of systemic acquired resistance			
	10	GO:0045454: cell redox homeostasis			
	12	GO:0010469: regulation of signalling receptor activity			
	30	GO:0006879: cellular iron ion homeostasis			
Stress response	1	GO:0010200: response to chitin			
	8	GO:0061408: positive regulation of transcription from RNA polymerase II promoter in response to heat stress			
	5	GO:0034605: cellular response to heat			
	11	GO:0006355: regulation of transcription, DNA-templated			
	19	GO:0000165: MAPK cascade			
	25	GO:0006970: response to osmotic stress			
	20	GO:0080167: response to karrikin			
	24	GO:0042542: response to hydrogen peroxide			
	2	GO:0006955: immune response			
	3	GO:0009611: response to wounding			
	4	GO:0009607: response to biotic stimulus			
	6	GO:0009723: response to ethylene			
	7	GO:0009626: plant-type hypersensitive response			
	27	GO:0046686: response to cadmium ion			
	28	GO:0009739: response to gibberellin			
	29	GO:0009651: response to salt stress			
	15	GO:0010039: response to iron ion			
	16	GO:0070413: trehalose metabolism in response to stress			
	17	GO:0035556: intracellular signal transduction			
	18	GO:0009617: response to bacterium			
	21	GO:0006073: cellular glucan metabolic process			
	22	GO:0009738: abscisic acid-activated signalling pathway			
	23	GO:0010167: response to nitrate			

Akivi (Aki), lyophilized (BL) and fresh (BF) Bacillus UdG treatments

ID: GO term assigned identifier

White space means not GO term

*Smaller values denote higher uniqueness. Red (0.7-0.8), orange (0.8-0.9), yellow (0.9-1.0)

From the cluster named “regulation of defense response”, the GO term regulation of jasmonic acid mediated signalling pathway was shared by all treatments. Whereas the GO term regulation of defense response was shared by Aki and BF, regulation of systemic acquired resistance was unique for Aki. From the cluster named “stress response”, the GO terms response to osmotic stress, response to karrikin, and response to hydrogen peroxide were unique for *Bacillus* (BF and BL), while the GO terms immune response and plant-type hypersensitive response, and response to wounding, biotic stimulus and ethylene were unique for Aki.

The upregulated genes (Log_2_(FC) > 1.4) related to the GO terms included in “regulation of defense response” and “stress response” clusters are shown in **Table 2** (**Supplementary Tables 7**, **8**). Interestingly, some upregulated DEGs were also unique for each treatment (Aki and *Bacillus*). After *Bacillus* treatment, one gene related to the regulation of jasmonic acid, and several genes related to transcription factors, chaperones, enzymes as catalase, PR protein with antimicrobial activity, abscisic acid receptor, and cold induced protein were upregulated. However, after Akivi treatment, two genes related to the regulation of SAR, one defense response related gene, three genes related to the response to chitin, and one gene related to PR protein with antimicrobial activity were upregulated.

**Table 2:** Upregulated genes included in the GO terms that belong to regulation and stress response groups after treatments of cv. Garnacha Blanca grapevine leaves with Akivi (Aki), lyophilized (BL) and fresh (BF) *Bacillus* UdG.

Description	Gene ID	GO ID	Aki	BF	BL
Enzymes					
Leucoanthocyanidin dioxygenase	VIT_13s0067g01020	9			
Trehalose 6-phosphate synthase	VIT_17s0000g08010; VIT_01s0026g00280	16			
Trehalose-phosphatase	VIT_12s0028g01670	16			
Xyloglucan endotransglucosylase/hydrolase	VIT_11s0052g01280; VIT_05s0062g00250; VIT_01s0026g00200	21			
Catalase	VIT_00s0698g00010	27			
Proteins that mediate the attachment of integral membrane proteins to the cytoskeleton					
Ankyrin repeat	VIT_14s0081g00370	13			
Ankyrin repeat	VIT_05s0165g00010; VIT_14s0081g00360	13			
Transcriptional regulators/Transcriptional factors					
Jasmonate ZIM domain-containing protein 8	VIT_10s0003g03790	9, 13			
Cold induced protein	VIT_17s0000g08010	27			
Zinc finger (C2H2 type) family	VIT_13s0019g00480	1			
Myb domain protein 14	VIT_05s0049g01020	1, 27, 29			
Salt tolerance homolog2	VIT_03s0038g00340	1, 20			
WRKY DNA-binding protein 33	VIT_08s0058g00690	1, 5, 25, 29			
Heat shock transcription factor C1	VIT_11s0016g03940	5, 8			
Modulators and regulators of related defense responses and cell death program					
NIM1	VIT_07s0005g02070; VIT_01s0011g03430	14			
NSL1 (necrotic spotted lesions 1)	VIT_01s0011g05950	2, 7			
Abscisic acid receptor PYL1 RCAR12	VIT_13s0067g01940	22			
Plant peptide growth factors.					
Phytosulfokines PSK1	VIT_08s0007g03870	12			
DNA replication					
DNA mismatch repair protein MSH3	VIT_00s0388g00030	26			
Iron storage and transport proteins					
Ferritin	VIT_08s0058g00440, VIT_08s0058g00430, VIT_08s0058g00410	15, 18, 24, 30			
Metal-nicotianamine transporter YSL1	VIT_02s0025g02510	15			
Chaperones (HSP)					
Heat shock protein 18.2 kDa class II	VIT_12s0035g01910	24, 29			
Heat shock protein 17.6 kDa class I	VIT_13s0019g03160	24, 29			
HSP (HSP26.5-P) 26.5 kDa class P	VIT_00s0992g00020	24, 29			
Pathogenesis related proteins					
Pathogenesis protein 10	VIT_05s0077g01570	22			
Pathogenesis protein 10	VIT_05s0077g01600	4			
Unknown					
unknown	VIT_09s0002g03340	27			

Go ID: GO term assigned identifier (**Table 1**)

**Figure 5:**
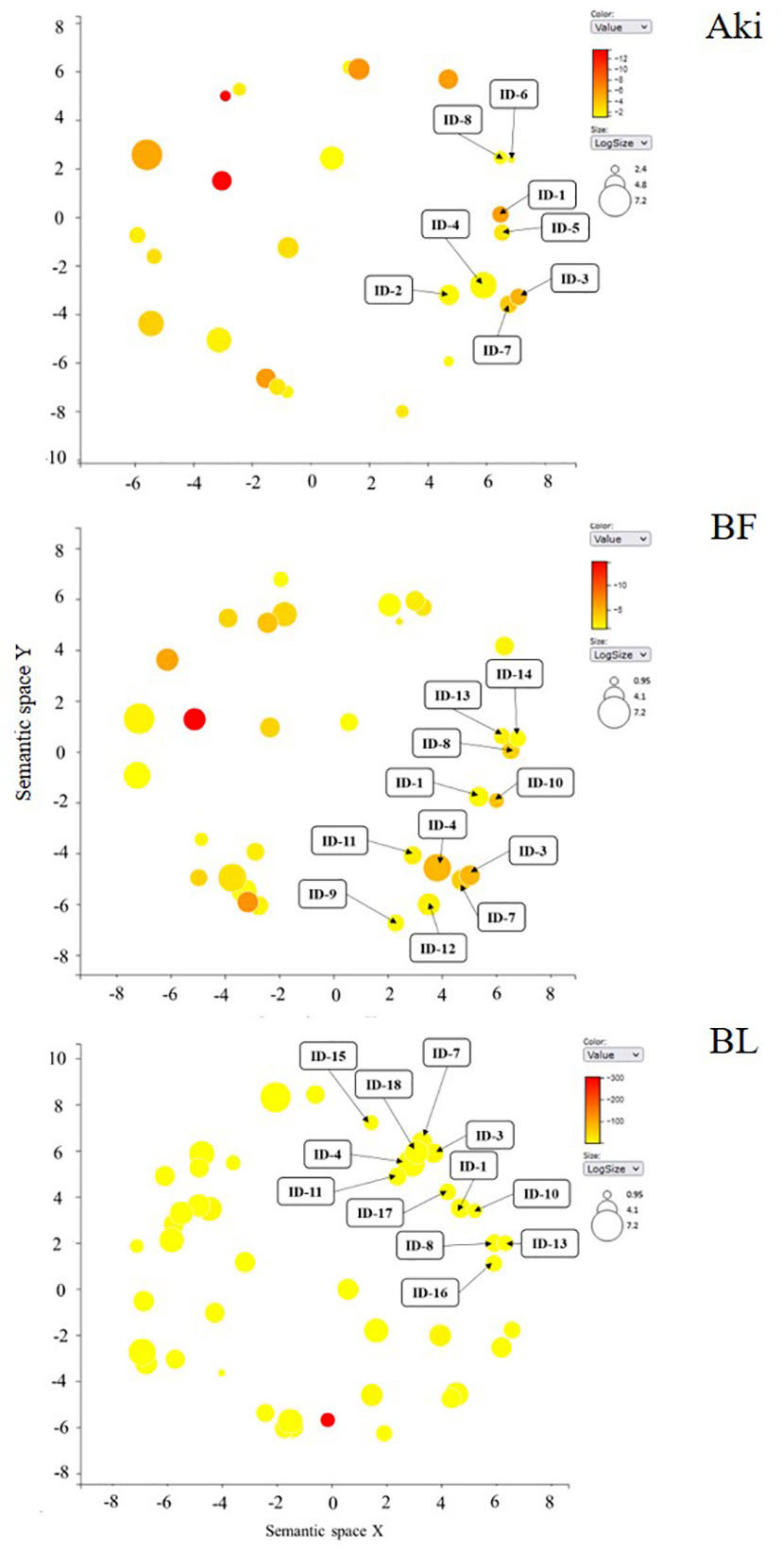
REVIGO graphs of downregulated GO term clusters (regulation of defense response and stress response) in leaves of cv. Garnacha Blanca grapevine included in biological process category after treatments with Akivi (Aki), lyophilized (BL) and fresh (BF) *Bacillus* UdG. ID: identification of GO terms associated with **Table 3**.

Some of the downregulated GO terms from BP category were arranged in a cluster related to plant defense response, namely “stress-related response” (**Figure 5**). This cluster include 18 GO terms related to regulation of cellular cycle and cell population proliferation, plant development, metabolic processes and their regulation, stress response, defense and response to stimuli and signal transduction (**Table 3**). The GO terms related to cellular cycle, stress and stimuli response, metabolic processes regulation and signal transduction were shared by the three treatments. However, GO terms related to plant development, defense response and metabolic processes were unique for *Bacillus* treatments.

**Table 3:** Representative groups (clusters) of downregulated GO terms of biological processes obtained with REVIGO and associated to plant defense responses, after treatments of cv. Garnacha Blanca grapevine leaves with Akivi (Aki), lyophilized (BL) and fresh (BF) *Bacillus* UdG.

			Uniqueness*		
Representative group	ID	GO ID: GO Term	Aki	BF	BL
Stress-related response	3	GO:0045787: positive regulation of cell cycle			
	4	GO:0006355: regulation of transcription			
	8	GO:0009414: response to water deprivation			
	7	GO:0008284: positive regulation of cell population proliferation			
	1	GO:0009734: auxin-activated signaling pathway			
	10	GO:0010017: red or far-red light signaling pathway			
	13	GO:0009744: response to sucrose			
	11	GO:0045910: negative regulation of DNA recombination			
	2	GO:0043086: negative regulation of catalytic activity			
	5	GO:0007178: transmembrane receptor protein serine/threonine kinase signaling pathway			
	6	GO:0071249: cellular response to nitrate			
	9	GO:0009909: regulation of flower development			
	12	GO:0043085: positive regulation of catalytic activity			
	14	GO:0046686: response to cadmium ion			
	15	GO:0010112: regulation of systemic acquired resistance			
	16	GO:0009627: systemic acquired resistance			
	17	GO:0000076: DNA replication checkpoint signaling			
	18	GO:0045893: positive regulation of transcription			

Akivi (Aki), lyophilized (BL) and fresh (BF) Bacillus UdG treatments

ID: GO term assigned identifier

White space means not GO term

* Smaller values denote higher uniqueness. Purple (0.7-0.8), blue (0.8-0.9), clear blue (0.9-1.0)

The downregulated genes (Log_2_(FC) < 1.4) related to the GO terms included in “stress-related response” cluster are shown in **Table 4** (**Supplementary Table 9**). Interestingly, after both Aki and *Bacillus* treatments, some DEGs related to cellular cycle were downregulated. In particular, six cyclin proteins and one annexin protein were downregulated for Aki treatment. While four cyclin proteins were downregulated for *Bacillus* treatments.

**Table 4:** Downregulated genes included in the GO terms that belong to stress-related response groups after treatments of cv. Garnacha Blanca grapevine leaves with Akivi (Aki), lyophilized (BL) and fresh (BF) *Bacillus* UdG.

Gene description	Gene ID	GO ID	Aki	BF	BL
Transcription factor related to auxin signalling pathway					
IAA31	VIT_05s0020g01070	1			
Proteins that control the cell cycle by activating cyclin-dependent kinase (CDK)/Cycle regulators					
Cyclin delta-3 (CYCD3_1)	VIT_18s0001g09920				
*Cyclin D3_2*	VIT_03s0180g00040	2, 3, 6, 7			
*Cyclin CYCB1_2*	VIT_06s0009g02090	2, 3, 6, 7			
*Cyclin B-type*	VIT_08s0040g00930	2, 3, 6, 7			
*Cyclin 1b (CYC1b)*	VIT_13s0067g01420	2, 3, 6, 7			
*Cyclin-dependent protein kinase regulator CYCB2_4*	VIT_18s0001g14170	2, 3, 6, 7			
Cyclin B2;4	VIT_03s0038g02800	3, 7			
Cyclin delta-2	VIT_03s0091g01060	3, 7			
Cyclin A1	VIT_18s0001g02060	3, 7			
Cyclin-dependent protein kinase CYCB3	VIT_19s0085g00690	3, 7			
Annexin ANN4	VIT_00s0131g00080	3,8			
Protein kinase WEE1	VIT_07s0104g01740	17			
Proteins that join DNA to form nucleosomes					
Histone H4	VIT_06s0004g04370; VIT_13s0019g00780; VIT_13s0019g00800	8			
Histone H1	VIT_07s0005g01060; VIT_07s0141g00730; VIT_14s0081g00500	11			
Receptors like-Kinases (RLK)					
Proline extensin-like receptor kinase 1 (PERK1)	VIT_01s0127g00670	3			
Receptor protein kinase	VIT_05s0020g01690	3			
DNA replication and repair					
ATP-dependent DNA helicase RecQ	VIT_01s0010g02590	3, 7			
Origin recognition complex subunit 5	VIT_01s0011g04400	13			
Origin recognition complex subunit 4	VIT_17s0000g01960	13			
DNA mismatch repair protein	VIT_01s0011g03440	15			
B ZipDNA binding proteins/Transcription factors/Zinc finger proteins					
BZIP protein HY5 (HY5)	VIT_04s0008g05210	10			
BZIP protein HY5 (HY5)	VIT_05s0020g01090	10			
BZIP transcription factor BZIP6	VIT_00s0541g00020	18			
AP2/ERF domain containing protein	VIT_08s0007g08150	18			
NAC Secondary wall thickening promoting factor1	VIT_02s0025g02710	18			
Late meristem identity1 HB51/LMI1	VIT_08s0007g04200	18			
Homeodomain leucine zipper protein HB-1	VIT_01s0026g01550	18			
Homeobox-leucine zipper protein HB-7	VIT_15s0048g02870	18			
Constans 2 (COL2)	VIT_14s0083g00640	9			
Zinc knucle	VIT_01s0010g01670	17			
Lipid Transfer Proteins (LTP)					
DIR1 (defective IN induced resistance 1)	VIT_00s0333g00050	16			
Protease inhibitor/seed storage/lipid transfer protein (LTP)	VIT_08s0007g01370	16			
Unknown					
unknown	VIT_04s0008g04200	5			
unknown	VIT_04s0023g03760	5			
unknown	VIT_07s0129g00200	3, 7			
unknown	VIT_13s0067g02560	18			

Go ID: GO term assigned identifier (**Table 3**)

Moreover, genes related to plant growth and development, such as transcriptional factors and zinc finger proteins, DNA replication, and two lipid transfer protein (LTP) that intervene in systemic acquired resistance SAR were downregulated after *Bacillus* treatments.

Considering different stress responses, after Aki treatment two genes connected with receptor like kinases that intervene in plant innate immunity were downregulated, while transcription factors to several stresses and abiotic stresses were downregulated after *Bacillus* treatments.

#### 3.3.2 KEGG pathway analysis of DEGs

KEGG pathway analysis was performed to evaluate the biological mechanisms influenced by the Aki, BL, and BF treatments. Few pathways were associated with DEGs affected by the treatments and none was shared between Akivi and *Bacillus* treatments **(** **Supplementary Table 10** **)**.

The following pathways “Glutathione metabolism”, “Linoleic acid metabolism”, “alpha-Linolenic acid metabolism”, “Terpenoid backbone biosynthesis”, “beta-Alanine metabolism”, and “Fatty acid degradation” were triggered after Aki treatment. Whereas “Starch and sucrose metabolism” was triggered by both BL and BF treatments, “Ribosome biogenesis in eukaryotes” was exclusively triggered by BF treatment.

The pathway “Porphyrin and chlorophyll metabolism” was reduced after Aki treatment, while “DNA replication” was reduced after both BL and BF treatments. “Cysteine and methionine metabolism” was reduced after BL treatment; while “Fructose and mannose metabolism”, “Phenylpropanoid biosynthesis”, and “Starch and sucrose metabolism” were reduced after BF treatment.

### 3.4 Gene marker candidates on grapevine

#### 3.4.1 Selection of DEGs

A total of 27 DEGs were selected since their expression level was modified due to Aki, BL and BF treatments according to the results of RNA-seq analysis (**Table 5**).

**Table 5:** Selected Differentially Expressed Genes (DEGs) on cv. Garnacha Blanca grapevine leaves after treatment with Akivi (Aki) and lyophilized *Bacillus* UdG (BL).

Code	Gene ID	Log_2_ (FC)	FC	FDR	vCOST Description
Akivi					
**A1**	VIT_12s0059g02600	4.96	31.18	5.01E-15	Receptor protein kinase RK20-1
**A2**	VIT_06s0004g03350	3.46	11.03	9.18E-24	Lateral organ boundaries protein 1
**A3**	VIT_05s0077g00520	3.17	8.99	7.76E-11	Gibberellin 2-oxidase
**A4**	VIT_17s0000g00200	3.23	9.40	1.58E-17	Ethylene-responsive transcription factor ERF114
**A5**	VIT_08s0058g00970	2.39	5.23	1.67E-17	Cationic peroxidase
**A6**	VIT_12s0055g01010	3.04	8.23	1.38E-28	Peroxidase
**A7**	VIT_00s0372g00040	2.74	6.68	1.68E-08	1,8-cineole synthase, chloroplast
**A8**	VIT_04s0023g02240	2.83	7.11	7.56E-56	S-adenosyl-L-methionine:salicylic acid carboxyl methyltransferase
**A9**	VIT_12s0034g01140	2.07	4.21	5.28E-21	Plastocyanin domain-containing protein
**A10**	VIT_19s0090g00660	2.01	4.03	1.03E-32	Lipase GDSL
**A11**	VIT_03s0088g00810	1.88	3.67	3.94E-16	Pathogenesis-related protein 1 precursor (PRP 1)
**A12**	VIT_07s0005g06090	1.67	3.19	9.06E-19	Pore-forming toxin-like protein Hfr-2
Bacillus					
**B1**	VIT_16s0022g00860	5.23	37.41	1.25E-28	Invertase/pectin methylesterase inhibitor
**B2**	VIT_06s0004g07210	5.45	43.58	4.78E-65	CCT motif constans-like
**B3**	VIT_16s0100g00740	4.26	19.16	2.49E-15	unknown
**B4**	VIT_14s0068g01160	2.91	7.53	5.22E-12	Cytokinin-repressed protein CR9
**B5**	VIT_00s1490g00010	-2.46	0.18	9.93E-38	5’-adenylylsulfate reductase (APR1)
**B6**	VIT_13s0064g01370	3.08	8.43	4.30E-07	Polygalacturonase inhibiting protein 1 PGIP1
**B7**	VIT_09s0002g04280	3.14	8.81	2.33E-47	Dynein light chain LC6, flagellar outer arm
**B8**	VIT_03s0091g00310	2.96	7.80	6.23E-16	Indole-3-acetic acid-amido synthetase GH3.8
**B9**	VIT_01s0011g01980	2.47	5.52	3.91E-22	fasciclin arabinogalactan-protein (FLA21)
**B10**	VIT_01s0026g02740	2.64	6.25	9.54E-28	unknown
**B11**	VIT_08s0058g00430	1.82	3.52	1.32E-02	ferritin
**B12**	VIT_10s0116g00530	1.96	3.89	1.07E-30	Thiazole biosynthetic enzyme, chloroplast (ARA6)
**B13**	VIT_00s0480g00060	1.49	2.81	7.91E-20	Polyphenol oxidase [Vitis vinifera]
**B14**	VIT_07s0031g02610	2.98	7.92	3.48E-14	NAC domain containing protein 2
**B15**	VIT_13s0067g02130	2.50	5.64	6.67E-10	Dehydration-induced protein (ERD15)

FC, fold change

FDR, false discovery rate

From the 12 DEGs highly triggered by Aki treatment, eight genes are related to defense response (A1, A3, A4, A5, A6, A7, A11, and A12). Specifically, two genes are involved in detoxification of reactive oxidative species (A5 and A6); two genes are related to hormone signalling pathway (A3, and A4), one gene is involved in biosynthesis of secondary metabolites (A7), and one gene is a marker of SAR response (A11). From the 15 DEGs highly triggered by BL and BF treatments, four genes are involved in defense response (B1, B6, B8, and B14).

#### 3.4.2 Validation of selected DEGs by RT-qPCR

Standard curves of the 27 DEGs showed R-squared values above 0.99 and, in general, amplification efficiencies above 90%, except for three DEGs (A1, A3 and A4) that showed slightly lower efficiencies above 80% (**Supplementary Table 3**). The expression levels of the 27 DEGs within the NTC samples on the ‘Garnacha Blanca’ experiment were stable showing FC values close to 1 (**Table 6**). The selected DEGs were upregulated after Aki (12) and BL treatments (14) with significant differences in comparison with the NTC, with the exception of B5 gene that was downregulated.

**Table 6:** Expression levels of the selected DEGs influenced by treatments of cvs. Garnacha Blanca, Garnacha Tinta, and Macabeo with Akivi (Aki) and lyophilized *Bacillus* UdG (BL).

	‘Garnacha Blanca’				‘Garnacha Tinta’				‘Macabeo’			
DEGs	NTC	Aki			NTC	Aki			NTC	Aki		
**A1**	1.29	14.37	± 3.96	*	1.29	4.86	± 4.91		1.11	4.46	± 1.73	*
**A2**	1.08	10.12	± 0.79	*	1.00	1.88	± 0.71	*	1.02	1.58	± 0.50	*
**A3**	1.05	5.64	± 0.57	*	1.03	1.43	± 0.25	*	1.04	1.74	± 0.81	*
**A4**	1.12	3.27	± 0.38	*	1.02	2.22	± 0.72	*	1.02	3.20	± 1.19	*
**A5**	1.05	8.01	± 1.07	*	1.02	1.55	± 0.32	*	0.99	1.68	± 0.30	
**A6**	1.05	9.11	± 0.44	*	1.09	1.39	± 0.49		1.05	1.16	± 0.80	
**A7**	1.06	3.39	± 0.61	*	1.06	1.65	± 0.59	*	1.10	1.10	± 0.60	
**A8**	1.08	4.71	± 1.71	*	1.05	2.94	± 1.90	*	1.16	1.78	± 1.27	
**A9**	1.06	4.56	± 1.14	*	1.01	2.79	± 2.32	*	1.01	0.44	± 0.21	*
**A10**	1.01	3.15	± 0.72	*	1.00	1.69	± 0.53	*	1.01	0.92	± 0.29	
**A11**	1.01	3.24	± 0.96	*	1.12	1.44	± 0.38		1.02	0.69	± 0.40	
**A12**	1.10	3.54	± 0.40	*	1.01	9.96	± 3.41	*	1.01	2.23	± 0.68	*
DEGs	NTC	BL			NTC	BL			NTC	BL		
**B1**	1.16	58.79	± 23.12	*	1.06	14.51	± 3.64	*	1.01	4.07	± 1.64	*
**B2**	1.05	22.43	± 3.57	*	1.03	3.19	± 0.34	*	1.02	4.05	± 1.17	*
**B3**	1.06	7.98	± 2.92	*	1.00	2.21	± 0.17	*	1.00	2.02	± 0.19	*
**B4**	1.15	5.49	± 1.84	*	1.09	0.77	± 0.20		0.90	1.63	± 0.13	
**B5**	1.04	0.22	± 0.07	*	1.02	0.50	± 0.18	*	1.09	3.03	± 0.58	*
**B6**	1.01	5.17	± 1.71	*	1.01	1.48	± 0.15	*	1.04	0.54	± 0.08	*
**B7**	1.29	6.86	± 1.57	*	1.04	1.39	± 0.25	*	1.07	0.71	± 0.12	*
**B8**	1.03	4.10	± 0.67	*	1.01	1.38	± 0.24		1.08	2.43	± 0.21	*
**B9**	1.09	8.30	± 3.71	*	1.01	5.07	± 1.22	*	1.06	2.76	± 0.59	*
**B10**	1.07	7.66	± 1.43	*	1.01	3.17	± 0.73	*	1.01	1.26	± 0.38	
**B11**	1.15	4.71	± 1.45	*	1.02	1.16	± 0.44		1.07	7.18	± 1.72	*
**B12**	1.01	4.68	± 0.90	*	1.01	3.76	± 0.54	*	1.03	8.59	± 1.48	*
**B13**	1.00	4.32	± 0.62	*	1.02	2.84	± 0.28	*	1.02	2.07	± 0.51	*
**B14**	1.12	5.96	± 1.32	*	1.04	3.15	± 0.76	*	1.22	2.50	± 0.22	*
**B15**	1.03	6.26	± 1.58	*	1.04	2.66	± 0.58	*	1.03	0.92	± 0.27	

DEG functions are indicated in **Supplementary Table 2**. Data correspond to RT-qPCR. The relative expression level of each gene was calculated by the comparative critical threshold (ΔΔCt) method using the non-treated control samples (NTC) as the calibrator and UQB gene as internal control for data normalization. Data mean the fold change (2^-ΔΔCt^) ± confidence interval and significant differences according to REST2009 Software are represented by *.

**Figure 6:**
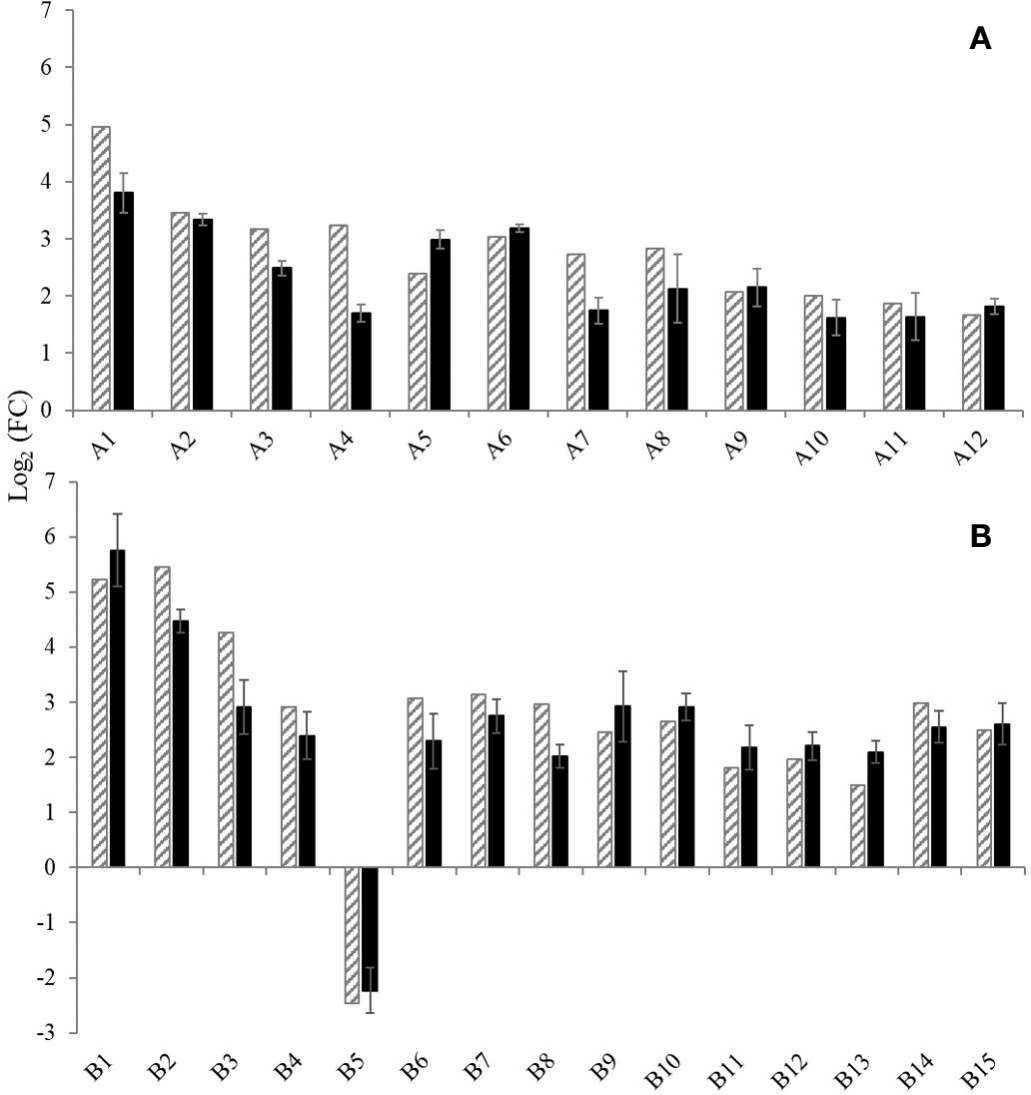
Expression levels of twenty-seven genes selected for validation of the RNA-Seq data by RT-qPCR. The gene expression was analysed after treatments with Akivi **(A)** and lyophilized *Bacillus* UdG **(B)**. RNA-seq (stripped bars) and RT-qPCR (black bars) analysis. Gene functions are indicated in **Supplementary Table 2**. RT-qPCR data are shown as the mean of Log_2 (_FC_)_of three biological replicates, where FC is the fold-change value and was calculated as 2^-ΔΔCt^ using non- treated control (NTC) samples as the calibrator and UBQ gene for data normalization. Error bars mean confidence interval of three biological replicates.

Moreover, the relative expression levels of the 27 DEGs on cv. Garnacha Blanca obtained by RT-qPCR and RNA-seq analysis were highly consistent for both Aki and BL treatments (**Figure 6**). That was confirmed by Pearson correlation test that showed high correlation coefficient values, 0.729 and 0.938 for Aki and BL, respectively, and statistical significances with p-values<0.05 (**Supplementary Figure 5**). Therefore, the 27 DEGs that were previously selected by RNA-seq analysis were validated by RT-qPCR on grapevine cv. Garnacha Blanca.

#### 3.4.3 Expression of validated DEGs in the three grapevine cultivars

The expression levels of the 27 DEGs were subjected to RT-qPCR using samples from experiments performed with two other grapevine cultivars, namely Garnacha Tinta and Macabeo. Within the NTC samples of the ‘Garnacha Tinta’ and ‘Macabeo’ experiments, the expression levels of the 27 DEGs were stable showing fold change values close to 1 (**Table 6**).

**Figure 7:**
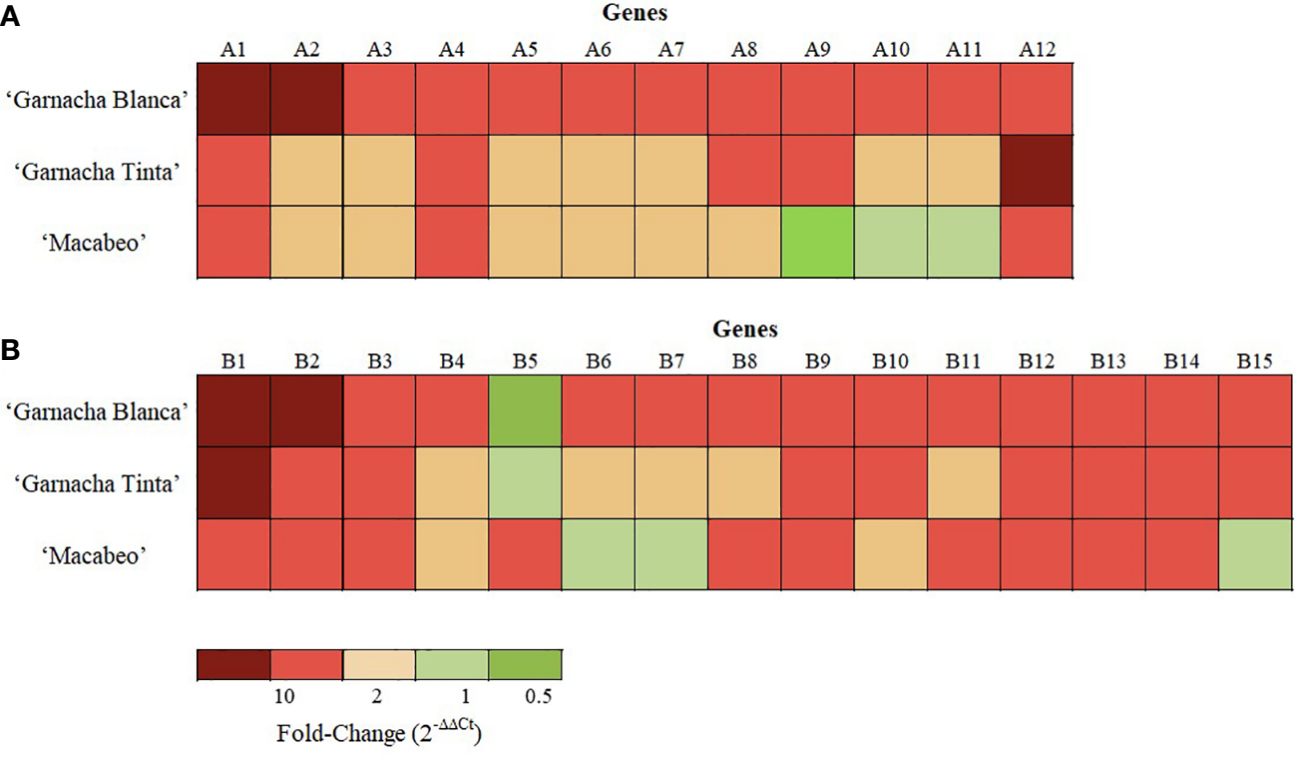
Transcriptional pattern of DEGs after treatments of grapevine cvs. Garnacha Blanca, Garnacha Tinta, and Macabeo with Akivi **(A)** or lyophilized *Bacillus* UdG **(B)**. The fold change was assessed by the ΔΔCt method. The UBQ gene was used as the internal control for data normalization. The ΔCt of the non-treated control (NTC) samples was defined as the calibrator. Three independent biological replicated were performed. Gene functions are indicated in **Supplementary Table 2**.

Concerning the 12 selected DEGs by Akivi treatment, nine (A2, A3, A4, A5, A7, A8, A9, A10, and A12) and six genes (A1, A2, A3, A4, A9, and A12) in ‘Garnacha Tinta’ and ‘Macabeo’, respectively, showed differential expression levels with statistical significance compared to the NTC (regardless of the FC value) (**Table 6**). After Akivi treatment, the A1, A4, and A12 genes were upregulated on the three grapevine cultivars (**Figure 7**). However, the A1 gene showed a FC value of 4.86 without significant differences with the NTC on ‘Garnacha Tinta’. Whereas the A9 gene was upregulated on ‘Garnacha Blanca’ and ‘Garnacha Tinta’, this gene was downregulated on ‘Macabeo’. In the case of A8 gene, despite it was upregulated on ‘Garnacha Blanca’ and ‘Garnacha Tinta’, its gene expression was not affected on ‘Macabeo’. Seven genes, namely, A2, A3, A5, A6, A7, A10 and A11, were only upregulated on ‘Garnacha Blanca’, while the expression pattern of these genes was not affected on ‘Garnacha Tinta’ and ‘Macabeo’. Therefore, the expression pattern after Akivi treatment was quite similar in the ‘Garnacha Blanca’ and ‘Garnacha Tinta’ (5 out of 12 genes were upregulated). However, the expression pattern obtained on ‘Macabeo’ differed from ‘Garnacha Blanca’ and ‘Garnacha Tinta’ since only 3 out of 12 (A1, A4, A12) genes were upregulated on all cultivars tested. In particular, three genes (A9, A10 and A11) showed FC below 1 only on ‘Macabeo’, being A9 downregulated.

In relation to the 15 selected DEGs by BL treatment, twelve genes showed differential expression levels with statistical significance compared to the NTC within ‘Garnacha Tinta’ (B1, B2, B3, B5, B6, B7, B9, B10, B12, B13, B14, and B15) and ‘Macabeo’ (B1, B2, B3, B5, B6, B7, B8, B9, B11, B12, B13, and B14) (regardless of the FC value) (**Table 6**). After BL treatment, B1, B2, B3, B9, B12, B13, and B14 genes were upregulated on the three grapevine cultivars (**Figure 7**). The only gene that was downregulated on ‘Garnacha Blanca’ was B5, which was unaltered and upregulated on ‘Garnacha Tinta’ and ‘Macabeo’, respectively. Three genes, namely B4, B6, and B7, were only upregulated on ‘Garnacha Blanca’, while they were unaltered (FC between 0.5-2) on ‘Garnacha Tinta’ and ‘Macabeo’. In the case of B8 and B11 genes, their expression levels were upregulated on both ‘Garnacha Blanca’ and ‘Macabeo’, while their expression levels were not affected on ‘Garnacha Tinta’. Two genes, namely B10 and B15, were upregulated on both ‘Garnacha Blanca’ and ‘Garnacha Tinta’, while their expressions were not affected on ‘Macabeo’. Therefore, the expression pattern after BL treatment was quite similar in the ‘Garnacha Blanca’ and ‘Garnacha Tinta’ (9 out of 15 genes were upregulated). Similar results were also observed comparing expression patterns in ‘Garnacha Blanca’ and ‘Macabeo’ (9 out of 15 genes were upregulated) despite B5 gene was clearly upregulated on ‘Macabeo’ and downregulated on ‘Garnacha Blanca’. However, the expression pattern obtained on ‘Macabeo’ differed from ‘Garnacha Tinta’ since a smaller number of genes (7 out of 15) shared the same upregulation transcriptional pattern.

### 3.5 Metabolite concentrations

Foliar Aki and BL treatments slightly influenced some of the mineral nutrient concentrations in the grapevine leaves, but not enough to affect plant development in any of the three cultivars (**Supplementary Table 11**).

Phytohormones, organic acids (OA) and total phenolic compounds concentrations were compared for each treatment (Aki and BL) with the NTC (**Table 7**).

**Table 7:** Phytohormone, organic acids, and total phenolic contents in leaves of grapevine cvs. Garnacha Blanca, Garnacha Tinta, and Macabeo treated with Akivi (Aki) and lyophilized *Bacillus* UdG (BL) and water (NTC).

	‘Garnacha Blanca’						‘Garnacha Tinta’						‘Macabeo’					
	NTC		Aki		BL		NTC		Aki		BL		NTC		Aki		BL	
Phytohormone																		
**JA**	4.39	± 1.65	4.83	± 1.21	6.15	± 1.24	4.18	± 1.74	5.54	± 1.48	10.68	± 0.72*	5.75	± 0.73	6.95	± 1.70	5.85	± 2.25
**MeJA**	3.71	± 0.26	4.95	± 0.24*	5.12	± 0.55*	4.98	± 0.23	4.76	± 0.16	4.31	± 0.13	4.41	± 0.34	4.13	± 0.22	4.00	± 0.33
**SA**	375	± 78	553	± 75	321	± 68	215	± 52	654*	± 147	375	± 67	131	± 17	218	± 26	159	± 34
**ACC**	8.86	± 0.28	10.44	± 0.48	10.65	± 0.65	11.06	± 0.65	10.23	± 0.38	11.36	± 0.33	12.69	± 0.67	11.54	± 1.13	12.13	± 0.55
**ABA^1^**	1.51	± 0.20	0.94		1.54		6.15	± 0.99	7.37	± 0.39	5.71	± 0.60	21.25	± 3.48	21.55	± 1.05	17.96	± 0.71
**GA1&4**	8.39	± 1.07	31.05	± 5.96*	18.29	± 2,77	9.49	± 2.47	6.02	± 4.34	6.70	± 0.29	27.34	± 5.57	4.13*	± 1.60	7.85*	± 5.43
Organic acid																		
**Oxalic**	4.47	± 0.50	3.75	± 0.95	3.28	± 0.66	3.38	± 0.32	3.54	± 0.90	5.35	± 1.59	2.33	± 0.05	1.76	± 0.42	2.80	± 0.38
**Tartaric**	15.51	± 0.65	15.23	± 0.61	16.30	± 0.52	19.45	± 0.40	17.48	± 0.20	17.20*	± 1.20	17.13	± 1.20	16.43	± 0.30	15.02	± 0.55
**Malic**	1.48	± 0.24	1.87	± 0.38	1.78	± 0.13	1.34	± 0.20	2.02	± 0.24	1.39	± 0.14	0.92	± 0.16	0.88	± 0.18	1.18	± 0.29
**Oxoglutaric**	346	± 63	413	± 96	631*	± 21	667	± 21	674	± 30	671	± 21	155	± 33	88*	± 4	78*	± 11
**Total phenolic**	200	± 25	359	± 18	395*	± 61	876	± 35	912	± 51	808	± 19	656	± 52	679	± 79	984*	± 46

JA: jasmonic acid; MeJA: methyl jasmonate; SA: salicylic acid; ACC: ethylene precursor; ABA: abscisic acid; and GA1&4: Gibberellins A1 and A4.

^1^ ABA concentration values in ‘Garnacha Blanca’ was at the limit of detection and only one value was detected for the treatment modalities (Aki and BL), thus, they were not included in statistical analysis. The following concentrations correspond to phytohormone (ng/g FW), organic acid (mg/g FW, except for oxoglutaric acid in µg/g FW), and total phenolic (µg gallic acid equivalent/g PF). Results are means ± standard deviation (n=3 biological replicates). Significant differences according to the Tukey test (parametric tests) or the Dunn test (non-parametric tests) between treatment (Aki or BL) and NTC are represented by asterisks (*).

Regarding Aki treatment, no phytohormones were significantly influenced in the same way among the three grapevine cultivars. The SA tended to present higher levels after Aki treatment in ‘Garnacha Blanca’ and ‘Macabeo’ and was significantly enhanced in ‘Garnacha Tinta’. The GAs showed an opposite pattern being their levels significantly increased in ‘Garnacha Blanca’, but reduced in ‘Macabeo’. The MeJA in ‘Garnacha Blanca’ was significantly enhanced after Aki treatment. Although all the studied phytohormones, with the exception of JA, tended to be enhanced after Aki treatment in ‘Garnacha Blanca’; JA, ACC, and ABA were not significantly influenced by Aki treatment in any of the three cultivars.

BL treatment significantly enhanced JA in ‘Garnacha Tinta’ and MeJA in ‘Garnacha Blanca’. Oppositely, GAs were significantly reduced after BL treatment in ‘Macabeo’. However, neither SA, ACC, nor ABA were significantly influenced by BL treatment in any of the three cultivars.

After BL or Aki treatment, phytohormone content changed without a clear pattern and the establishment of a defense signalling triggering mechanism was not possible to infer from our data.

It is worth to mention that ABA global values detected in ‘Macabeo’ are higher than the values detected in the two ‘Garnacha’ varieties.

Four OA were identified in the leaves of the three grapevine cultivars: oxalic, tartaric, malic, and oxoglutaric (**Table 7**). Aki and BL treatments caused a significant reduction in oxoglutaric acid in ‘Macabeo’, but BL significantly increased the amount of this organic acid in ‘Garnacha Blanca’. BL treatment also reduced the level of tartaric acid in ‘Garnacha Tinta’. The rest of organic acids were not altered.

Total phenolic compounds concentration was significantly enhanced after BL treatment in ‘Macabeo’ and in ‘Garnacha Blanca’. Aki treatment only tend to increase the level of total phenolic compounds.

## 4 Discussion

**Figure 8:**
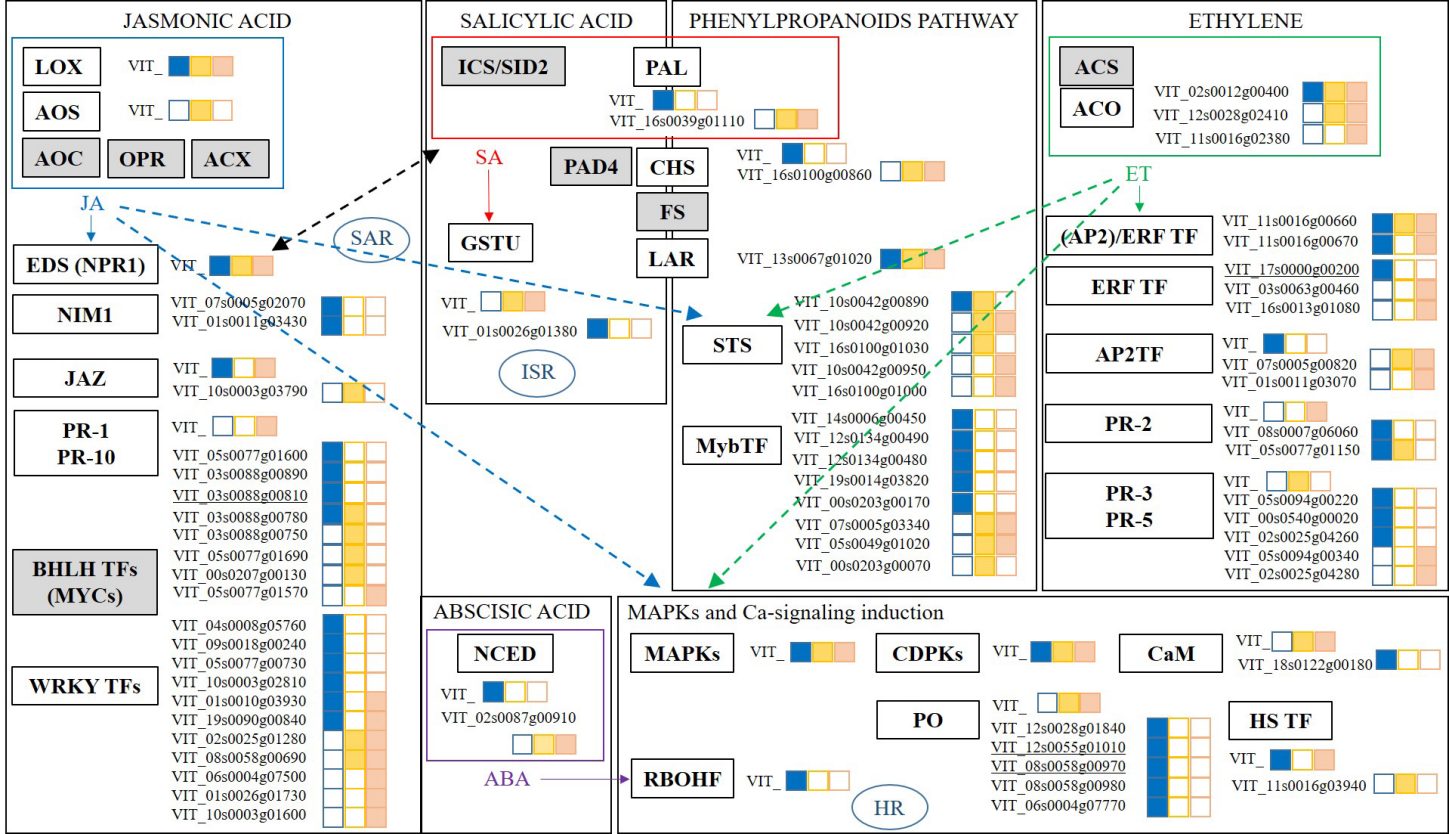
Scheme of main pathways related to plant defense response: Jasmonic Acid (JA); Salicylic Acid (SA); Ethylene (ET); Abscisic Acid (ABA); phenylpropanoids pathway; and mitogen activated protein kinases, Ca^2+^ signalling induction (MAPKs). DEGs results are presented from RNA-seq analysis of grapevine leaves treated with the botanical extract (Aki, blue) and the microbial product (BF, yellow; or BL, orange). Complete DEGs transcript codes are written when the differential expression is above Log (FC) > 1.4; only VIT_ is written otherwise. DEGs highly impacted by one of the treatments are underlined. Gene groups from the different pathways are indicated, the box is white coloured when transcripts related to the genes’ groups were found, the box is grey coloured otherwise. JA and ET interactions with other pathways are represented with arrows. Black arrow represents JA and SA crosstalk. LOX, LipOXygenase; AOS, Allene Oxide Synthases; AOC, Allene Oxide Cyclase; OPR, OPDA Reductase; ACX, Acetyl-CoA oXidase; EDS1/NPR1, Enhanced Disease Susceptibility/Non-expressor of Pathogen Related genes 1; NIM1, Non-Inducible Immunity 1; SAR, Systemic Acquired Resistance; JAZ, JAsmonate-Zim domain; PR, Pathogenesis Related proteins; BHLH TFs, Helix Loop Helix TFs, WRKY TFs, Transcription Factors with domain WRKY. ICS/SID2, IsoChorismate Synthase; PAL, Phenylalanine Ammonia Lyase; PAD4, PhytoAlexin Deficient 4; FS, Flavonoid Synthase; LAR, LeucoAnthocyanicin Dioxygenase; GSTs, Glutatione-S-Tranferase; ISR, Induced Systemic Resistance; STS, STilbene Synthase; MyB TF, MyB Transcription Factors. ACS; 1-Aminocyclopropane-1-Carboxylate Synthase; ACO, 1- Aminocyclopropane-1-Carboxylate Oxidase; ERF TF, (AP2)/ERF TF/AP2TF; Ethylene Response Factors Transcription Factors; PR, Pathogenesis Related proteins. NCED, 9-Cis-Epoxycarotenoid Dioxygenase. MAPKs, Mitogen Activated Protein Kinases; CDPKs, Ca^2+^ DePendent Kinases; CaM, CalModulin; RBOHF, Respiratory Burst Oxidase Homologue protein F; PO, PerOxidases; HS TF, Heat Shock Transcription Factors.

The response of grapevines to foliar treatments with the botanical extract Akivi (Aki) and the beneficial microorganism *Bacillus* UdG (fresh, BF or lyophilized, BL) was investigated. Among the environmentally friendly compounds considered as PPPs by the European Union legislation, beneficial microorganisms and natural substances (i.e. plant extracts) are included. *Bacillus* UdG and Akivi were used in this study representing each modality. Previous studies have reported the features and potential of both biocontrol products (Mora et al., 2011; Mora et al., 2015; Ramos et al., 2022). **Figure 8** shows a scheme as a summary of the genes related to the main plant defense response pathways (Jasmonic Acid, JA; Salicylic Acid, SA; Ethylene, ET; Abscisic Acid, ABA; phenylpropanoids pathway; and mitogen activated protein kinases and Ca^2+^ signalling induction, MAPKs) whose expression levels were influenced by the treatments. Interestingly, the two *Bacillus* UdG treatments, both BF and BL triggered the same pathways. However, BF and BL did not always trigger the same genes of the above-mentioned pathways. These results underline the importance of the product formulation since it may determine its efficacy and mode of action. Only Aki and BL treatments will be discussed hereafter.

Gene transcription related to JA biosynthesis was slightly influenced by either Aki and BL treatments since lipoxygenases (LOX) related genes did not show overexpression with Log_2_(FC) values higher than 1.4. However, phytohormone concentrations related to JA defense pathway were affected in cvs. Garnacha Blanca and Tinta. Specifically, MeJA concentration was significantly higher after Aki and BL treatments in ‘Garnacha Blanca’ and JA concentration was doubled after BL treatment in ‘Garnacha Tinta’. In agreement with metabolite contents, several genes regulated by JA pathway were upregulated by the treatments. Namely, the expression level of genes related to non-inducible immunity 1 (NIM1) were upregulated by Aki treatment and the expression level of a gene related to enhanced disease susceptibility (EDS1) and to nonexpressor of pathogenesis-related genes 1 (NPR1) was slightly upregulated by both treatments. Moreover, Aki and BL treatments upregulated the expression of several genes involved in different transcription factors with WRKYs domain (WRKYs TFs) and pathogenesis related proteins (PR). Interestingly, one of the PR related genes (VIT_03s0088g00810) was highly influenced by Aki treatment. Therefore, JA defensive pathway seemed to be triggered by both treatments. This is in agreement with the essential role of JA as a phytohormone involved in the regulation of defense gene expression (Rienth et al., 2019), such as EDS1, NPR1, or NIM1 related genes (Ochsenbein et al., 2006; Pajerowska-Mukhtar et al., 2013; Backer et al., 2019). These results enlightened the link between PR upregulated genes and WRKYs TFs but they did not underline the bond with transcriptional regulators jasmonate-zim domain (JAZ) intermediate related genes as previously described (Kazan and Manners, 2012; Guerreiro et al., 2016).

The expression level of genes related to SA pathway were not clearly affected by neither Aki nor BL treatments. However, the measured concentration of SA phytohormone in grapevine leaves treated with Aki tended to be higher than in leaves treated with NTC or BL on all three cultivars, especially in ‘Garnacha Tinta’ in which significant differences were observed. It could be explained by the upregulation of the expression of some genes related to EDS1, NIM1, and NPR1 already commented above in JA paragraph. Actually the mentioned genes are described as modulators that intervene in SA accumulation and they are produced in crosstalk between SA and JA pathways (Rustérucci et al., 2001; Zhu et al., 2011; Chen et al., 2021). Interestingly, the expression level of the glutathione-S-transferase (GST) related gene was also upregulated by Aki treatment. It is reported that SA is able to regulate several genes from GST family that are upregulated through SA pathway in treated plants with beneficial microorganisms resulting in ISR priming (Gullner et al., 2018). GST family enzymes are involved in detoxifying cytotoxic compounds and the process implies transmembrane transport (Burdziej et al., 2021), which is in agreement with these results since “transmembrane transport” GO term was influenced by Aki and BL treatments.

The biosynthesis of ET seemed to be triggered by both treatments through the upregulation of the expression level of 1-aminocyclopropane-1-carboxylate oxidase (ACO) related genes. Moreover, the concentration of the ET precursor 1-aminocyclopropane-1-carboxylic acid (ACC) tended to present higher levels in ‘Garnacha Blanca’ leaves after Aki and BL treatments. These results were in agreement with the upregulation of expression of genes related to response factors regulated by ET (ERF TF, (AP2)/ERF TF, AP2 TF) by both treatments. In addition, the expression level of one of the genes related to ERF TF was highly influenced by Aki treatment (VIT_17s0000g00200). Therefore, ET defensive pathway seemed to be triggered by both treatments. ET response factors are key regulators of JA, ET, and ABA pathways in response to biotic and abiotic stresses, activating PR genes, such as osmotins (PR-5), chitinases (PR-3) and β-1,3-glucanases (PR-2) (Mizoi et al., 2012; Bahieldin et al., 2016; Rienth et al., 2019), which were indeed upregulated after both Aki and BL treatments.

The ABA biosynthesis was triggered by BL treatment through the upregulation of 9-cis-epoxycarotenoid dioxygenase (NCED) related gene expression. In fact, ABA biosynthesis starts with carotenoids and involves NCED enzyme that is strongly upregulated by stress (Xiong and Zhu, 2003). ABA is involved in the response to water stress and particularly intervenes in stomatal closure (Catacchio et al., 2019; Postiglione and Muday, 2020). It is expected variability in water stress response between grapevine cultivars because the two ‘Garnacha’ are more resistant to drought than ‘Macabeo’ (Mirás-Avalos and Araujo, 2021). These results are consistent with ABA measured concentrations that were twice or three times higher in ‘Macabeo’ than in the two ‘Garnacha’. Actually, ‘Macabeo’, which is less resistant to drought, is more likely to trigger water stress response involving ABA signaling. ABA is also involved in pathogen response signaling pathway and linked with SA, JA, and ET related genes regulation (Nishad et al., 2020). For instance, ABA biosynthesis induction by laminarin treatment triggered JA production in grapevine (Balestrini et al., 2020). However, ABA relation with JA-dependent related genes are closely linked with MYCs TF (Pieterse et al., 2014) but were not influenced by any of the treatments in the present study.

This study also underlined that the expression of a Phenylalanine Ammonia Lyase (PAL) and a Chalcone Synthase (CHS) related genes were upregulated after BL treatment, whereas the expression of one Leucoanthocyanidin dioxygenase (LAR) related gene was upregulated after both Aki and BL treatments. PAL, CHS, LAR, and flavonol synthase (FS) are key enzymes for biosynthesis of several secondary metabolites, such as phenylpropanoids, or phytoalexins isoflavonoids (Campos et al., 2003; Yonekura-Sakakibara et al., 2019). These enzymes are related to SA biosynthesis sharing PAL enzyme as showed in the results. Stilbene biosynthesis was also triggered by both treatments through the upregulation of Stilbene Synthase (STS) and Myb TF related gene expression. The transcriptomic results were in accordance with the total phenolic concentration in leaves, which tended to be higher after Aki and BL treatments in ‘Garnacha Blanca’ and statistically higher in ‘Macabeo’ after BL treatment. It is worth to mention that the phytohormones JA, MeJA, SA, ET, and ABA positively regulate stilbene biosynthesis (Dubrovina and Kiselev, 2017). In agreement with these results, JA and ET strongly trigger phenylpropanoids pathway, notably stilbene biosynthesis (Belhadj et al., 2008; Rienth et al., 2019).

The expression level of several genes related to Mitogen-Activated Protein Kinases (MAPKs) and Calcium ion (Ca^2+^) signaling pathways were slightly upregulated after Aki treatment and some of them after BL treatment as well, such as Ca^2+^ Dependent Kinases (CDPKs), Calmodulin (CaM), Respiratory Burst Oxydase Protein (RBOHF) and Heat Shock Transcription Factors (HS TFs) with an upregulation lower than Log_2_(FC) > 1.4. In addition, one CaM and several peroxidases (PO) related genes were clearly upregulated after Aki treatment, and two of them were highly upregulated by Aki treatment (VIT_12s0055g01010; VIT_08s0058g00970) and involved in hypersensitive response (HR). As no phytotoxicity was observed after the treatments, Aki treatment may prime HR to be faster in case of pathogen infection. A crosstalk is described between MAPKs, JA, SA, and ET pathways (Rasmussen et al., 2012; Jagodzik et al., 2018; Nishad et al., 2020) and it was confirmed in this study since all these pathways were upregulated by the treatments.

In addition, Aki and BL treatments had an effect on other metabolites, including the Oxoglutaric acid (2-OG) that showed higher concentration in ‘Garnacha Blanca’ leaves after BL treatment and lower concentration in ‘Macabeo’ leaves after Aki and BL treatments. The 2-OG is involved in gibberellin (GA), alkaloid and flavonoid biosynthesis (Puhl et al., 2008; Araújo et al., 2012). Indeed, it was reported that treating grapevine with a structural mimic of 2-OG (prohexadione-Ca) inhibit the enzyme and alter flavonoid biosynthesis (high amount of unusual flavonoids) (Puhl et al., 2008). This is in agreement with the obtained results since phenylpropanoids pathway was triggered by both Aki and BL treatments. Moreover, the GA content in leaves was also affected in the present study, being GA1 and GA4 concentrations higher after the treatments (Aki or BL) in ‘Garnacha Blanca’ and lower in ‘Macabeo’. However, the link between 2-OG and GA concentrations’ variations was not clear, that reinforce the hypothesis linking the 2-OG with flavonoid biosynthesis.

The concentration of Tartaric acid in grapevine leaves was also affected since lower concentrations were detected in ‘Garnacha Tinta’ leaves treated with BL. Grapevine presents a high concentration on tartaric acid and its biosynthesis occurs in leaves and berries. Tartaric acid was shown to be involved in various processes and abrupt changes in its biosynthesis were linked with oxidative burst as well as ascorbate/glutathione redox state in berries (Burbidge et al., 2021). More insight on this matter could give interesting results like a kinetics study of tartaric acid after BL treatment.

Grapevine response to Aki and BL treatments at transcripts and metabolic level indicate the ability of these products to trigger plant defense response. In fact, many transcripts related to defense responses were detected by the RNA-seq analysis of leaves. Some of the transcripts did not present differential expression, but others were highly affected by Aki treatment (VIT_17s0000g00200, VIT_08s0058g00970, VIT_12s0055g01010, and VIT_03s0088g00810) and were selected as DEGs markers candidates (A4, A5, A6, and A11, respectively). Considering all the upregulated transcripts in JA, ET, SA, and ABA pathways (**Figure 8**) and the higher concentrations of some phytohormones; the application of Aki and BL treatments to grapevine can stimulate several processes related to plant defense immune system like SAR. Particularly, the treatments upregulated JA, ET, and phenylpropanoid pathways. Moreover, Aki treatment seemed to trigger several genes involved with HR. Further investigations are necessary to identify the mode of action of the two biocontrol product candidates (Aki and BL).

These results also indicate that the treatments with Aki and the BL might prime a defense response through ISR. However, the study was designed to investigate the interaction between the biocontrol products and grapevine without pathogen infection. If the mode of action is priming ISR, the effect could be seen only with the presence of the pathogen attack (Van Wees et al., 2008; Pieterse et al., 2014; Esmaeel et al., 2020). Actually, a complex effect acting in two steps was observed on various biocontrol products, such as the *Rheum palmatum* plant extract (Godard et al., 2009), *Trichoderma harzianum* T39 (Perazzolli et al., 2011), and sulphated laminarin (Trouvelot et al., 2008), being this last one already used in vineyards against downy mildew. These products show plant defense stimulation activity through the induction of some genes immediately after the treatment and the reinforcement of the modulation of defense response through other genes after pathogen inoculation. The pathogen infection may trigger biocontrol product activity and different grapevine response as it was observed using transcriptomics in watermelon (*Citrullus lanatus*) roots treated with the beneficial microorganism candidate *B. velezensis* against *Fusarium oxysporum* fungal pathogen (Jiang et al., 2019). More insights on Aki and BL possible modes of action could be revealed through another investigation introducing pathogen inoculation in the study, such as *P. viticola*, *E. necator*, or *B. cinerea* and analyzing grapevine response to the treatment after pathogen infection. This new research could be more accurate by doing a sampling kinetics to study the plant response to both treatments and pathogen inoculation along time by transcriptomic and metabolomics approaches (Jiang et al., 2019).

From the twelve DEGs selected for Aki treatment eight are related to plant defense (A1, A3, A4, A5, A6, A7, A11, and A12). Some of them (A4, A5, A6, and A11) are involved in the main pathways related to plant defense response (**Figure 8**) and were discussed above. From the fifteen DEGs selected for BL treatment, four of them are related to plant defense (B1, B6, B8, and B14). In addition, another gene could be related to defense response (B12-VIT_10s0116g00530) as it is involved in thiazole biosynthesis. Thiazole is a precursor of thiamine that has been showed to be able to stimulate defense response (Boubakri et al., 2012; Boubakri et al., 2013). It is reported that thiamine is able to induce resistance to downy mildew defense response elicitation in leaves of ‘Chardonnay’ cultivated in greenhouse-controlled conditions. The elicited defense response included accumulation of hydrogen peroxide (H_2_O_2_), callose deposition in stomata cells, phenylpropanoid compounds accumulation (stilbenes, phenolic compounds, flavonoids and lignin) and hypersensitive response. Thiamine triggered several genes involved in defense response like PR genes (glucanase, chitinases, serine protease inhibitor, glutathione-S-transferase) and lipoxygenases pathway involved in JA biosynthesis (Boubakri et al., 2012; Boubakri et al., 2013). The high rate of DEGs highly impacted by the treatments and related to defense response is consistent with the transcripts analysis previously mentioned, and with the hypothesis that Aki and BL treatments could be able to induce resistance on grapevine.

Several DEGs markers presented stable overexpression after Aki (A1, A4 and A12 genes) and BL (B1, B2, B3, B9, B12, B13, and B14 genes) treatments in the three grapevine cultivars. Therefore, they could be considered as appropriate markers of Akivi and *Bacillus* treatments and could be used to test different doses and formulations of the biocontrol products in greenhouse-controlled conditions. Actually, defining treatment dose and formulation are crucial steps in product development that highly impact its efficacy. As observed in the present study, grapevine response is variable according to the studied cultivar as described other studies (Bota et al., 2016; Catacchio et al., 2019; Fasoli et al., 2019; Balestrini et al., 2020). Therefore, the identified markers are only robust for the three tested cultivars and should be tested on other cultivars to extend their use. The markers could also be tested in field conditions, but it could be difficult to detect an impact on transcriptome in field conditions due to vineyard biological variability (Balestrini et al., 2020).

## 5 Conclusion

Grapevine response to the Aki and BL treatments at transcripts and metabolites levels gave insights on modes of action of these biocontrol products that are under development. RNA sequencing analysis showed different gene expression patterns after foliar treatments with the biocontrol products compared with the NTC in ‘Garnacha Blanca’. Furthermore, RT-qPCR enabled the quantification of several selected genes (DEG) in three different cultivars. This information was complemented with metabolic analysis (phytohormones, phenols, and organic acids). Considering all the upregulated transcripts and enhanced metabolites concentrations related to JA, ET, and phenylpropanoids pathways, strong indication was found of grapevine defense induction by the treatments. However, further studies are necessary to confirm these first results and a kinetics study could be interesting. In addition, several DEGs markers were identified presenting a stable overexpression after the treatments (Aki or BL) in the three grapevine cultivars. They could be used as markers of activity of the products for further investigations.

## Data availability statement

The original contributions presented in the study are publicly available. This data can be found here: https://www.ncbi.nlm.nih.gov/geo/query/acc.cgi?acc=GSE211268.

## Author contributions

ML, EM, and EB obtained the financial support. MR, ND, ML, EM, and EB conceived and designed the research. MR, ND, ML, RT and EB conducted and performed the experiments. MR, ML, and EB analyzed the data. MR and ND wrote the first draft of the manuscript. ML, EM, and EB revised and edited the manuscript. All authors contributed to the article and approved the submitted version.
