# Supplementary material for: Grapevine response to a Dittrichia viscosa extract and a Bacillus velezensis strain
Source: Front Plant Sci. 2022 Dec 16;13:1075231. doi: 10.3389/fpls.2022.1075231 (PMC9803176; doi:10.3389/fpls.2022.1075231)
Supplement: Supplementary file 1 [file DataSheet_1.docx]

Supplementary Material

**Supplementary Table 1.** Concentration (ng µL^-1^), purity (Abs_260/280_ and Abs_260/230_ ratios) and integrity (RIN) of total RNA in samples of cv. Garnacha grapevine leaves obtained with five different protocols. Total RNA samples treated using the Spectrum™ Plant Total RNA Kit fulfilled the quantity and quality requirements to carry on RNA-sequencing (RNA-seq) analysis (RNA concentration above 200 ng µL^-1^, Abs_260/280_ ratio from 1.9 to 2.1, Abs_260/230_ ratio from 1.8 to 2.2, and RIN above 7).

| **RNA isolation protocol** | **Replicate** | **Spectrophotometric measurements**^6^ | | |  |
| --- | --- | --- | --- | --- | --- |
|  |  | **RNA concentration (ng µL^-1^)** | **Abs_260/280_** | **Abs_260/230_** | **RIN**^7^ |
| PureLink^®^ Plant RNA Reagent^1^ | R1 | 1138.0 | 2.01 | 1.85 | 8.10 |
|  | R2 | 553.1 | 1.99 | 1.59 | 3.40 |
|  | R3 | 454.4 | 1.92 | 1.52 | 2.80 |
| TRIzol™ Reagent^2^ | R1 | 85.4 | 0.79 | 0.14 | - |
|  | R2 | 62.8 | 0.76 | 0.13 | - |
|  | R3 | 75.6 | 0.78 | 0.13 | - |
| Spectrum™ Plant Total RNA Kit^3^ | R1 | 507.0 | 2.06 | 1.97 | 8.80 |
|  | R2 | 473.8 | 2.05 | 1.94 | 8.80 |
|  | R3 | 483.0 | 2.00 | 1.97 | 8.60 |
| CTAB 2-day extraction^4^ | R1 | 222.4 | 2.07 | 1.87 | 4.80 |
|  | R2 | 624.0 | 2.09 | 2.1 | 3.70 |
|  | R3 | 326.9 | 2.07 | 1.97 | 4.10 |
| CTAB 1-day extraction^5^ | R1 | 32.1 | 1.69 | 0.64 | - |
|  | R2 | 58.3 | 1.87 | 1.16 | - |
|  | R3 | 42.6 | 1.75 | 0.95 | - |
| ^1, 2^ Invitrogen^TM^, Life Technologies, Carlsbad, CA  ^3^ Sigma-Aldrich, Merck KGaA, Darmstadt, Germany  ^4^ Iandolino et al., 2004  ^5^ Mu et al., 2017  ^6^ Spectrophotometric measurements using NanoDrop ND-1000 Spectrophotometer (Thermo Fisher Scientific, Waltham, MA)  ^7^ RNA Integrity Number (RIN). RIN measurement was performed using an Agilent 2100 Bioanalyzer (Agilent technologies, USA) | | | | | |

**Supplementary Table 2.** Differentially Expressed Genes (DEGs) and endogenous genes primer sequences used in the present study. The optimized primer concentrations for qPCR analysis are also shown.

| **Code** | **Gene ID** | **Gene description** |  | **Forward primer sequence (5'-3')** |  | **Reverse primer sequence (5'-3')** | **[primer] (nM)** |
| --- | --- | --- | --- | --- | --- | --- | --- |
| **Endogenous genes for expression data normalization** | | | | | | | |
| E1 | EC922622 | Ubiquitin-conjugating enzyme (UBQ) |  | GAGGGTCGTCAGGATTTGGA |  | GCCCTGCACTTACCATCTTTAAG | 300 |
| E2 | XM_002281110.1 | Vacuolar ATPase subunit G (VAG) |  | TTGCCTGTGTCTCTTGTTC |  | TCAATGCTGCCAGAAGTG | 300 |
| **Selected DEGs impacted by *Bacillus* treatment** | | | | | | | |
| B1 | VIT_16s0022g00860 | Invertase/pectin methyl esterase inhibitor |  | GCTGCAAGAAATGTGGAATGC |  | TCGACTCTTGTGACTTTGTTTTCC | 100 |
| B2 | VIT_06s0004g07210 | CCT motif constans-like |  | CAAGTGCCAGACACCATCCT |  | ACCAGCACCGCACATACTTT | 100 |
| B3 | VIT_16s0100g00740 | unknown |  | CCAGACACGTCTGACTCCAC |  | CAGCTCCACGGTAACTCCTG | 300 |
| B4 | VIT_14s0068g01160 | Cytokinin-repressed protein CR9 |  | AGAAGCCTGCTTGGCAGATT |  | CCGGAACACCGTTTTTGCAT | 300 |
| B5 | VIT_00s1490g00010 | 5'-adenylylsulfate reductase (APR1) |  | AAGTTCAGGGCTTGGTGAGG |  | GGGTCTCACTTCTCACACGG | 300 |
| B6 | VIT_13s0064g01370 | Polygalacturonase inhibiting protein 1 PGIP1 |  | AGGCGAGTTTCATGGAGCAG |  | GGAATTTTCCCACACAACCTGT | 300 |
| B7 | VIT_09s0002g04280 | Dynein light chain LC6, flagellar outer arm |  | GGGGAAAATAAGGTGCGGGA |  | ACAGGGCCCTCATCACAATG | 300 |
| B8 | VIT_03s0091g00310 | Indole-3-acetic acid-amido synthetase GH3.8 |  | TCGCCCTTATGACCCCTACA |  | AGGACTTGTTTGCGCTCGTA | 100 |
| B9 | VIT_01s0011g01980 | Fasciclin arabinogalactan-protein (FLA21) |  | TTGCATTGTGCAGCAAACGA |  | GGATGCCACGTGGTCCATAA | 100 |
| B10 | VIT_01s0026g02740 | Unknown |  | GGTGACTGCACCAGTGATTG |  | AGTGGCTGCTCTAACAACCT | 300 |
| B11 | VIT_08s0058g00430 | Ferritin |  | CCTCTCATCTGCATCTTTCTCGT |  | TCCCCTGACGACCCTAAGAG | 300 |
| B12 | VIT_10s0116g00530 | Thiazole biosynthetic enzyme, chloroplast (ARA6) |  | TGGCCAGGCCTAATGTGAAG |  | AACCAAAGCCCAGTTGGTGA | 300 |
| B13 | VIT_00s0480g00060 | Polyphenol oxidase |  | GCTTTTCTTCCCTTTCCACCG |  | CGGCATTTGCATTCCAGGAG | 100 |
| B14 | VIT_07s0031g02610 | NAC domain containing protein 2 |  | CTCTCCAAGGGACCGCAAAT |  | AATTCCGACCGTCTTGGGTC | 300 |
| B15 | VIT_13s0067g02130 | Dehydration-induced protein (ERD15) |  | TATCGGACGGTGGAGGACTT |  | AGCCAGTAATCGCGAAACCA | 300 |
| **Selected DEGs impacted by Akivi treatment** | | | | | | | |
| A1 | VIT_12s0059g02600 | Receptor protein kinase RK20-1 |  | TGTGTCACTGAGGCAACCAA |  | TCGTACCAAATGATCGCTCC | 100 |
| A2 | VIT_06s0004g03350 | Lateral organ boundaries protein 1 |  | GCGAGCTTCAAGCGCAATTA |  | AGGTTTGCTTGCTGGCATTG | 300 |
| Continue | | | | | | | |

**Table 1.** (continued)

| **Code** | **Gene ID** | **Gene description** |  | **Forward primer sequence (5'-3')** |  | **Reverse primer sequence (5'-3')** | **[primer] (nM)** |
| --- | --- | --- | --- | --- | --- | --- | --- |
| A3 | VIT_05s0077g00520 | Gibberellin 2-oxidase |  | AATGGGAGGTTTGTGAGCGT |  | GAAGGCCTCTCAGGTGTGAC | 300 |
| A4 | VIT_17s0000g00200 | Ethylene-responsive transcription factor ERF114 |  | AAGTGGGCAGCTGAGATACG |  | TAGGCAAGTGCAGCATCCTC | 300 |
| A5 | VIT_08s0058g00970 | Cationic peroxidase |  | CTCCGCTTGACACCAAAAGC |  | ACTTGAGAATCCGTGGAGCC | 300 |
| A6 | VIT_12s0055g01010 | Peroxidase |  | CGCAAAGTGTGCTCTGCAAT |  | AGTGCATGTGAGAAGTTACGGA | 100 |
| A7 | VIT_00s0372g00040 | 1,8-cineole synthase, chloroplast |  | CAAGGCACAGATGGATGGGT |  | GCAGCATCTCCTTCTGGTGT | 100 |
| A8 | VIT_04s0023g02240 | S-adenosyl-L-methionine: salicylic acid carboxyl methyltransferase |  | GGGACACCAGTTACGCAGAA |  | GGTCCAGAAGAACAGCCCAA | 300 |
| A9 | VIT_12s0034g01140 | Plastocyanin domain-containing protein |  | TAGCCCTTCGGCTCACAATG |  | AATAGTTGGCCCCCTTCACC | 300 |
| A10 | VIT_19s0090g00660 | Lipase GDSL |  | AATTGGGCTTACCATCCGCA |  | TCAAAGATTCCGGCACCTCC | 300 |
| A11 | VIT_03s0088g00810 | Pathogenesis-related protein 1 precursor (PRP 1) |  | CAATGGAGGGTGGTTCGTCA |  | CACCATGCTCTAACAGTACCCA | 100 |
| A12 | VIT_07s0005g06090 | Pore-forming toxin-like protein Hfr-2 |  | TTTACGTTGGCGTGAACTGC |  | CAAGGAAGGGGGATTCGACC | 300 |

**Supplementary Table 3.** Standard curves and amplification efficiencies of selected DEGs and endogenous genes. Efficiency was calculated using the following formula: E = (10^(-1⁄a)^ -1)x100; where “a” is the slope of the curve.

| **Gene code** | **Slope** | **Linearity (R^2^)** | **Efficiency (%)** |
| --- | --- | --- | --- |
| **Endogenous genes for expression data normalization** | | | |
| **UBQ** | -3.1975 | 0.9982 | 94.5319 |
| **VAG** | -3.1984 | 0.9980 | 94.5736 |
|  |  |  |  |
| **Selected DEGs impacted by *Bacillus* treatment** | | | |
| **B1** | -3.3549 | 0.9990 | 98.6422 |
| **B2** | -3.2400 | 0.9992 | 96.4637 |
| **B3** | -3.1767 | 0.9984 | 93.5608 |
| **B4** | -3.2187 | 0.9995 | 95.5042 |
| **B5** | -3.3248 | 0.9965 | 99.8803 |
| **B6** | -3.1607 | 0.9958 | 92.8020 |
| **B7** | -3.1415 | 0.9956 | 91.8774 |
| **B8** | -3.2086 | 0.9991 | 95.0432 |
| **B9** | -3.2204 | 0.9968 | 95.5814 |
| **B10** | -3.2200 | 0.9996 | 95.5632 |
| **B11** | -3.1472 | 0.9992 | 92.1535 |
| **B12** | -3.1988 | 0.9995 | 94.5921 |
| **B13** | -3.1957 | 0.9995 | 94.4486 |
| **B14** | -3.3611 | 0.9990 | 98.3909 |
| **B15** | -3.1398 | 0.9997 | 91.7948 |
|  |  |  |  |
| **Selected DEGs impacted by Akivi treatment** | | | |
| **A1** | -2.9448 | 0.9922 | 81.4345 |
| **A2** | -3.2068 | 0.9923 | 94.9606 |
| **A3** | -2.9788 | 0.9977 | 83.3765 |
| **A4** | -2.9636 | 0.9920 | 82.5159 |
| **A5** | -3.2492 | 0.9984 | 96.8728 |
| **A6** | -3.2250 | 0.9988 | 95.7898 |
| **A7** | -3.2345 | 0.9990 | 96.2175 |
| **A8** | -3.1898 | 0.9991 | 94.1745 |
| **A9** | -3.2784 | 0.9997 | 98.1509 |
| **A10** | -3.1796 | 0.9997 | 93.6973 |
| **A11** | -3.1849 | 0.9994 | 93.9457 |
| **A12** | -3.2421 | 0.9996 | 96.5573 |
| UBQ: Ubiquitin-conjugating enzyme  VAG: Vacuolar ATPase subunit G | | | |

**Supplementary Table 4.** Trimming of the total reads obtained by RNA-seq of each sample: Akivi (Aki), lyophilized (BL) and fresh (BF) *Bacillus* UdG, and non-treated control (NTC). Four biological replicates (R1, R2, R3 and R4).

| Sample | Before Trimming | After Trimming |
| --- | --- | --- |
| Aki_R1 | 38,686,326 | 32,735,444 (84.62%) |
| Aki_R2 | 31,168,690 | 26,280,264 (84.32%) |
| Aki_R3 | 45,077,410 | 38,346,848 (85.07%) |
| Aki_R4 | 42,671,630 | 36,470,692 (85.47%) |
| BF_R1 | 35,932,354 | 30,911,962 (86.03%) |
| BF_R2 | 39,632,304 | 34,292,640 (86.53%) |
| BF_R3 | 30,960,456 | 26,219,334 (84.69%) |
| BF_R4 | 37,725,956 | 32,086,976 (85.05%) |
| BL_R1 | 44,957,978 | 38,463,562 (85.55%) |
| BL_R2 | 33,369,744 | 28,770,018 (86.22%) |
| BL_R3 | 33,800,350 | 29,312,548 (86.72%) |
| BL_R4 | 33,260,154 | 28,436,820 (85.50%) |
| NTC_R1 | 43,724,406 | 36,870,722 (84.33%) |
| NTC_R2 | 35,748,642 | 30,338,146 (84.87%) |
| NTC_R3 | 38,252,254 | 32,522,744 (85.02%) |
| NTC_R4 | 38,341,052 | 32,965,052 (85.98%) |

**Supplementary Table 5.** Mapping of the paired reads (fragments) obtained by RNA-seq of each sample: Akivi (Aki), lyophilized (BL) and fresh (BF) *Bacillus* UdG, and non-treated control (NTC). Four biological replicates (R1, R2, R3 and R4).

| Sample | Input paired reads | Unique Mapping | Multi Mapping | Unmapped | Assigned to genes |
| --- | --- | --- | --- | --- | --- |
| Aki_R1 | 16,367,722 | 15,513,853 (94.78%) | 366,741 (2.24%) | 487,128 (2.98%) | 13,934,098 (85.13%) |
| Aki_R2 | 13,140,132 | 12,443,289 (94.70%) | 310,816 (2.37%) | 386,027 (2.94%) | 11,078,501 (84.31%) |
| Aki_R3 | 19,173,424 | 18,135,605 (94.59%) | 452,430 (2.36%) | 585,389 (3.05%) | 16,330,627 (85.17%) |
| Aki_R4 | 18,235,346 | 17,297,878 (94.86%) | 396,665 (2.18%) | 540,803 (2.97%) | 15,920,944 (87.31%) |
| BF_R1 | 15,455,981 | 14,719,273 (95.23%) | 314,501 (2.03%) | 422,207 (2.73%) | 13,665,530 (88.42%) |
| BF_R2 | 17,146,320 | 16,289,530 (95.00%) | 355,456 (2.07%) | 501,334 (2.92%) | 14,754,625 (86.05%) |
| BF_R3 | 13,109,667 | 12,479,648 (95.19%) | 277,620 (2.12%) | 352,399 (2.69%) | 11,255,908 (85.86%) |
| BF_R4 | 16,043,488 | 15,137,197 (94.35%) | 325,128 (2.03%) | 581,163 (3.62%) | 13,828,936 (86.20%) |
| BL_R1 | 19,231,781 | 18,296,618 (95.14%) | 395,252 (2.06%) | 539,911 (2.81%) | 16,991,936 (88.35%) |
| BL_R2 | 14,385,009 | 13,609,023 (94.61%) | 301,182 (2.09%) | 474,804 (3.30%) | 12,282,573 (85.38%) |
| BL_R3 | 14,656,274 | 13,857,455 (94.55%) | 314,967 (2.15%) | 483,852 (3.30%) | 12,522,586 (85.44%) |
| BL_R4 | 14,218,410 | 13,540,312 (95.23%) | 291,038 (2.05%) | 387,060 (2.72%) | 12,471,903 (87.72%) |
| NTC_R1 | 18,435,361 | 17,494,387 (94.90%) | 377,416 (2.05%) | 563,558 (3.06%) | 16,124,887 (87.47%) |
| NTC_R2 | 15,169,073 | 14,419,641 (95.06%) | 325,207 (2.14%) | 424,225 (2.80%) | 13,078,392 (86.22%) |
| NTC_R3 | 16,261,372 | 15,380,534 (94.58%) | 364,828 (2.24%) | 516,010 (3.17%) | 13,780,451 (84.74%) |
| NTC_R4 | 16,482,526 | 15,546,674 (94.32%) | 340,108 (2.06%) | 595,744 (3.61%) | 14,006,571 (84.98%) |

**Supplementary Table 6.** Gene Ontology (GO) terms influenced by treatments with Akivi (Aki), lyophilized (BL) and fresh (BF) *Bacillus* UdG compared to the non-treated control (NTC) on cv. Garnacha Blanca grapevine leaves. UP: upregulated, DOWN: downregulated. Criteria: Log_2_(FC) ≥ |1.4| (FDR significant) and four or more genes per GO term.

| **GO Category** | **Aki_UP** | **Aki_DOWN** | **BL_UP** | **BL_DOWN** | **BF_UP** | **BF_DOWN** |
| --- | --- | --- | --- | --- | --- | --- |
| Biological Process | 184/497 | 225/616 | 262/932 | 329/999 | 220/798 | 340/804 |
| Cell Component | 17/90 | 44/135 | 39/209 | 86/260 | 40/188 | 62/191 |
| Molecular Function | 135/390 | 139/404 | 151/613 | 204/624 | 121/538 | 225/518 |
| **Total Categories** | **336/977**  (34.4%) | **408/1155** (35.3%) | **452/1754** (25.8%) | **619/1883** (32.9%) | **381/1524** (25.0%) | **627/1513** (41.4%) |
| Selected GO terms according to the criteria / total significant GO terms | | | | | | |

**Supplementary Table 7.** Genes included in the GO terms that belongs to the group of defense response regulation and in which their expression pattern shows upregulation (Log_2_ (FC) ≥ 1.4) after treatments with Akivi (Aki), lyophilized (BL) and fresh (BF) *Bacillus* UdG.

|  |  |  |  | **Aki** | | **BF** | | **BL** | |  | **Other** |
| --- | --- | --- | --- | --- | --- | --- | --- | --- | --- | --- | --- |
| **ID** | **GO ID** | **GO Description** | **Gene ID** | **log_2_(FC)** | **FDR** | **log_2_(FC)** | **FDR** | **log_2_(FC)** | **FDR** | **vCOST Description** | **ID*** |
| 30 | GO:0006879 | cellular ion iron homeostasis | VIT_08s0058g00440 |  |  |  |  | 1,96 | 8,77E-04 | ferritin | 15, 18, 24 |
|  |  |  | VIT_08s0058g00430 |  |  |  |  | 1,82 | 1,32E-02 | ferritin | 15, 18, 24 |
|  |  |  | VIT_08s0058g00410 |  |  |  |  | 1,60 | 3,84E-03 | ferritin 1 (FER1) | 15, 18, 24 |
| 9 | GO:2000022 | regulation of JA mediated signalling pathway | VIT_13s0067g01020 | 1,98 | 5,40E-04 | 2,84 | 4,03E-11 | 2,32 | 1,49E-07 | Leucoanthocyanidin dioxygenase |  |
|  |  |  | VIT_10s0003g03790 |  |  | 1,83 | 2,44E-03 |  |  | Jasmonate ZIM domain-containing protein 8 | 13 |
| 12 | GO:0010469 | regulation of signalling receptor activity | VIT_18s0001g08760 | 1,81 | 1,42E-11 |  |  |  |  | *Phytosulfokines* |  |
| 13 | GO:0031347 | regulation of defense response | VIT_14s0081g00370 | 1,42 | 9,94E-06 | 1,85 | 3,59E-09 |  |  | Ankyrin repeat |  |
|  |  |  | VIT_05s0165g00010 |  |  | 5,41 | 9,83E-06 |  |  | Ankyrin repeat |  |
|  |  |  | VIT_10s0003g03790 |  |  | 1,83 | 2,44E-03 |  |  | Jasmonate ZIM domain-containing protein 8 | 9 |
|  |  |  | VIT_14s0081g00360 |  |  | 1,53 | 6,02E-13 |  |  | Ankyrin repeat |  |
| 14 | GO:0010112 | regulation of SAR | VIT_07s0005g02070 | 1,86 | 4,11E-24 |  |  |  |  | *NIMIN-1 ortologue* |  |
|  |  |  | VIT_01s0011g03430 | 1,76 | 3,64E-21 |  |  |  |  | *NIM-1-Intracting 2 like* |  |
| 26 | GO:0051096 | positive regulation of helixase activity | VIT_00s0388g00030 |  |  | 1.45 | 3,85E-01 | 2,32 | 8,58E-04 | DNA mismatch repair protein MSH3 |  |
| Akivi (Aki), lyophilized (BL) and fresh (BF) *Bacillus* UdG treatments | | | | | | | | | | | |
| ID: GO term assigned identifier. (*) the gene was also upregulated in this ID. Description in cursive means that other databases were used instead of vCOST. | | | | | | | | | | | |
| FDR, false discovery rate. Upregulated gene: log_2_(FC) ≥ 1.4 | | | | | | | | | | | |

**Supplementary Table 8.** Genes included in the GO terms that belongs to the group of stress response regulation and in which their expression pattern shows upregulation (Log_2_ (FC) ≥ 1.4) after treatments with Akivi (Aki), lyophilized (BL) and fresh (BF) *Bacillus* UdG.

|  |  |  |  | **Aki** | | **BF** | | **BL** | |  | **Other** |
| --- | --- | --- | --- | --- | --- | --- | --- | --- | --- | --- | --- |
| **ID** | **GO ID** | **GO Description** | **Gene ID** | **log_2_(FC)** | **FDR** | **log_2_(FC)** | **FDR** | **log_2_(FC)** | **FDR** | **vCOST Description** | **ID*** |
| 1 | GO:0010200 | Response to chitin | VIT_13s0019g00480 | 1,87 | 4,63E-05 |  |  |  |  | Zinc finger (C2H2 type) family |  |
|  |  |  | VIT_03s0038g00340 | 1,77 | 5,41E-10 | 1,86 | 8,79E-06 | 1,53 | 6,21E-07 | Salt tolerance homolog2 | 20 |
|  |  |  | VIT_05s0049g01020 |  |  | 1,69 | 9,99E-04 | 1,73 | 1,37E-03 | Myb domain protein 14 | 27, 29 |
|  |  |  | VIT_08s0058g00690 |  |  | 1,68 | 7,00E-07 | 2,12 | 1,08E-06 | WRKY DNA-binding protein 33 | 5, 25, 29 |
| 2 | GO:0006955 | Immune response | VIT_01s0011g05950 | 1,71 | 2,33E-06 |  |  |  |  | NSL1 (necrotic spotted lesions 1) | 7 |
| 4 | GO:0009607 | Response to biotic stimulus | VIT_05s0077g01600 | 1,61 | 1,21E-03 |  |  |  |  | Pathogenesis protein 10 [Vitis vinifera] |  |
| 5 | GO:0034605 | Celular response to heat | VIT_08s0058g00690 |  |  | 1,68 | 7,00E-07 | 2,12 | 1,08E-06 | WRKY DNA-binding protein 33 | 1, 25, 29 |
|  |  |  | VIT_11s0016g03940 |  |  | 1,49 | 1,73E-04 |  |  | Heat shock transcription factor C1 | 8 |
| 7 | GO:0009626 | Plant-type hypersensitive response | VIT_01s0011g05950 | 1,71 | 2,33E-06 |  |  |  |  | NSL1 (necrotic spotted lesions 1) | 2 |
| 8 | GO:0061408 | Positive regulation of transcription from RNA polymerase II promoter in response to heat stress | VIT_11s0016g03940 |  |  | 1,49 | 1,73E-04 |  |  | Heat shock transcription factor C1 | 5 |
| 15 | GO:0010039 | Response to iron ion | VIT_08s0058g00440 |  |  |  |  | 1,96 | 8,77E-04 | ferritin | 18, 24, 30 |
|  |  |  | VIT_08s0058g00430 |  |  |  |  | 1,82 | 1,32E-02 | ferritin | 18, 24, 30 |
|  |  |  | VIT_08s0058g00410 |  |  |  |  | 1,60 | 3,84E-03 | ferritin 1 (FER1) | 18, 24, 30 |
|  |  |  | VIT_02s0025g02510 |  |  |  |  | 1,52 | 2,54E-05 | Metal-nicotianamine transporter YSL1 |  |
| 16 | GO:0070413 | Trealose metabolism in respons to stress | VIT_17s0000g08010 |  |  |  |  | 2,06 | 4,40E-05 | Trehalose 6-phosphate synthase |  |
|  |  |  | VIT_12s0028g01670 |  |  |  |  | 1,93 | 6,43E-06 | Trehalose-phosphatase |  |
|  |  |  | VIT_01s0026g00280 |  |  |  |  | 1,72 | 3,72E-18 | Trehalose 6-phosphate synthase |  |
| Continue | | | | | | | | | | | |

**Supplementary Table 8.** (continued)

|  |  |  |  | **Aki** | | **BF** | | **BL** | |  | **Other** |
| --- | --- | --- | --- | --- | --- | --- | --- | --- | --- | --- | --- |
| **ID** | **GO ID** | **GO Description** | **Gene ID** | **log_2_(FC)** | **FDR** | **log_2_(FC)** | **FDR** | **log_2_(FC)** | **FDR** | **vCOST Description** | **ID*** |
| 18 | GO:0009617 | Response to bacterium | VIT_08s0058g00410 |  |  |  |  | 1,60 | 3,84E-03 | ferritin 1 (FER1) | 15, 24, 30 |
|  |  |  | VIT_08s0058g00430 |  |  |  |  | 1,82 | 1,32E-02 | ferritin | 15, 24, 30 |
|  |  |  | VIT_08s0058g00440 |  |  |  |  | 1,96 | 8,77E-04 | ferritin | 15, 24, 30 |
| 20 | GO:0080167 | Response to karritin | VIT_03s0038g00340 |  |  | 1,86 | 8,79E-06 | 1,53 | 6,21E-07 | Salt tolerance homolog2 | 1 |
| 21 | GO:0006073 | Cellular glucan metabolic process | VIT_11s0052g01280 |  |  |  |  | 2,68 | 9,56E-05 | *Xyloglucan endotransglucosylase/hydrolase* |  |
|  |  |  | VIT_05s0062g00250 |  |  |  |  | 1,91 | 2,28E-28 | Xyloglucan endotransglucosylase/hydrolase 15 |  |
|  |  |  | VIT_01s0026g00200 |  |  |  |  | 1,50 | 3,19E-13 | Xyloglucan endotransglucosylase/hydrolase 28 |  |
| 22 | GO:0009738 | Abscisic acid activated signalling pathway | VIT_13s0067g01940 |  |  |  |  | 1,66 | 4,14E-07 | Abscisic acid receptor PYL4 RCAR10 |  |
|  |  |  | VIT_05s0077g01570 |  |  |  |  | 1,60 | 3,19E-06 | *PR10* |  |
| 24 | GO:0042542 | Response to hydrogen peroxide | VIT_12s0035g01910 |  |  | 2,32 | 1,82E-03 |  |  | Heat shock protein 18.2 kDa class II | 29 |
|  |  |  | VIT_13s0019g03160 |  |  | 1,75 | 8,91E-04 |  |  | Heat shock protein 17.6 kDa class I | 29 |
|  |  |  | VIT_00s0992g00020 |  |  | 1,63 | 1,11E-22 | 1,44 | 2,48E-17 | Heat shock protein (HSP26.5-P) 26.5 kDa class P | 29 |
|  |  |  | VIT_08s0058g00440 |  |  |  |  | 1,96 | 8,77E-04 | ferritin | 15, 18, 30 |
|  |  |  | VIT_08s0058g00410 |  |  |  |  | 1,60 | 3,84E-03 | ferritin 1 (FER1) | 15, 18, 30 |
|  |  |  | VIT_00s0698g00010 |  |  |  |  | 1,60 | 9,15E-11 | Catalase |  |
|  |  |  | VIT_08s0058g00430 |  |  |  |  | 1,82 | 1,32E-02 | ferritin | 15, 18 |
| 25 | GO:0006970 | Response to osmotic stress | VIT_08s0058g00690 |  |  | 1,68 | 7,00E-07 | 2,12 | 1,08E-06 | WRKY DNA-binding protein 33 | 1, 5, 29 |
| Continue | | | | | | | | | | | |

**Supplementary Table 8.** (continued)

|  |  |  |  | **Aki** | | **BF** | | **BL** | |  | **Other** |
| --- | --- | --- | --- | --- | --- | --- | --- | --- | --- | --- | --- |
| **ID** | **GO ID** | **GO Description** | **Gene ID** | **log_2_(FC)** | **FDR** | **log_2_(FC)** | **FDR** | **log_2_(FC)** | **FDR** | **vCOST Description** | **ID*** |
| 27 | GO:0046686 | Response to cadmium ion | VIT_09s0002g03340 |  |  | 2,76 | 9,79E-04 |  |  | unknown |  |
|  |  |  | VIT_04s0079g00810 |  |  | 1,89 | 7,11E-03 |  |  | Cold induced protein |  |
|  |  |  | VIT_05s0049g01020 |  |  | 1,69 | 9,99E-04 |  |  | Myb domain protein 14 | 1, 5, 25 |
| 29 | GO:0009651: | Response to salt stress | VIT_12s0035g01910 |  |  | 2,32 | 1,82E-03 |  |  | Heat shock protein 18.2 kDa class II | 24 |
|  |  |  | VIT_13s0019g03160 |  |  | 1,75 | 8,91E-04 |  |  | Heat shock protein 17.6 kDa class I | 24 |
|  |  |  | VIT_05s0049g01020 |  |  | 1,69 | 9,99E-04 |  |  | Myb domain protein 14 | 1, 27 |
|  |  |  | VIT_08s0058g00690 |  |  | 1,68 | 7,00E-07 |  |  | WRKY DNA-binding protein 33 | 1, 5, 25 |
|  |  |  | VIT_00s0992g00020 |  |  | 1,63 | 1,11E-22 |  |  | Heat shock protein (HSP26.5-P) 26.5 kDa class P | 24 |
| Akivi (Aki), lyophilized (BL) and fresh (BF) *Bacillus* UdG treatments | | | | | | | | | | | |
| ID: GO term assigned identifier. (*) the gene was also upregulated in this ID. Description in cursive means that other databases were used instead of vCOST. | | | | | | | | | | | |
| FDR, false discovery rate. Upregulated gene: log_2_(FC) ≥ 1.4. | | | | | | | | | | | |

**Supplementary Table 9.** Genes included in the GO terms that belongs to the group of stress-related response regulation and in which their expression pattern shows downregulation (Log_2_(FC) ≥ -1.4) after treatments with Akivi (Aki), lyophilized (BL) and fresh (BF) *Bacillus* UdG.

|  |  |  |  | **Aki** | | **BF** | | **BL** | |  | **Other** |
| --- | --- | --- | --- | --- | --- | --- | --- | --- | --- | --- | --- |
| **ID** | **GO ID** | **GO Description** | **Gene ID** | **log_2_(FC)** | **FDR** | **log_2_(FC)** | **FDR** | **log_2_(FC)** | **FDR** | **vCOST Description** | **ID*** |
| 1 | GO:0009734 | Auxin-activated signaling pathway | VIT_05s0020g01070 |  |  |  |  | -2,04 | 8,48E-07 | IAA31 |  |
| 2 | GO:0043086 | Negative regulation of catalytic activity | VIT_18s0001g09920 | -1,40 | 3,67E-07 |  |  |  |  | Cyclin delta-3 (CYCD3_1) |  |
| *2, 3, 6 and 7* | *GO:0043086, GO:0045787, GO:0071249 and GO:0008284* | *Negative regulation of catalytic activity, Positive regulation of cell cycle, Cellular response to nitrate, and Positive regulation of cell population proliferation* | *VIT_03s0180g00040* | *-2,48* | *4,16E-08* |  |  |  |  | *Cyclin D3_2* |  |
|  |  |  | *VIT_06s0009g02090* | *-2,73* | *3,90E-03* |  |  |  |  | *Cyclin CYCB1_2* |  |
|  |  |  | *VIT_08s0040g00930* | *-1,55* | *1,49E-10* |  |  |  |  | *Cyclin B-type* |  |
|  |  |  | *VIT_13s0067g01420* | *-1,67* | *6,59E-06* |  |  |  |  | *Cyclin 1b (CYC1b)* |  |
|  |  |  | *VIT_18s0001g14170* | *-1,51* | *2,61E-05* |  |  |  |  | *Cyclin-dependent protein kinase regulator CYCB2_4* |  |
| 3 | GO:0045787 | Positive regulation of cell cycle | VIT_03s0038g02800 |  |  | -1,89 | 6,37E-09 | -1,93 | 1,55E-12 | Cyclin B2;4 | 7 |
|  |  |  | VIT_03s0091g01060 |  |  |  |  | -1,49 | 1,85E-11 | Cyclin delta-2 | 7 |
|  |  |  | VIT_07s0129g00200 |  |  |  |  | -1,65 | 2,50E-02 | unknown | 7 |
|  |  |  | VIT_18s0001g02060 |  |  | -1,46 | 1,13E-04 | -2,00 | 1,26E-10 | Cyclin A1 | 7 |
|  |  |  | VIT_19s0085g00690 |  |  | -1,40 | 8,44E-04 | -1,42 | 2,16E-04 | Cyclin-dependent protein kinase CYCB3 | 7 |
|  |  |  | VIT_01s0127g00670 | -2,60 | 3,69E-07 |  |  |  |  | Proline extensin-like receptor kinase 1 (PERK1) |  |
|  |  |  | VIT_05s0020g01690 | -1,79 | 5,21E-06 |  |  |  |  | Receptor protein kinase |  |
| 5 | GO:0007178 | Transmembrane receptor protein serine/threonine kinase signaling patway | VIT_04s0008g04200 | -1,86 | 1,99E-05 |  |  |  |  | unknown |  |
|  |  |  | VIT_04s0023g03760 | -1,49 | 9,27E-05 |  |  |  |  | unknown |  |
| 7 | GO:0008284 | Positive regulation of cell population proliferation | VIT_01s0010g02590 |  |  |  |  | -1,47 | 5,14E-03 | *ATP-dependent DNA helicase RecQ* |  |
|  |  |  | VIT_03s0038g02800 |  |  | -1,89 | 6,37E-09 | -1,93 | 1,55E-12 | Cyclin B2;4 | 3 |
|  |  |  | VIT_03s0091g01060 |  |  |  |  | -1,49 | 1,85E-11 | Cyclin delta-2 | 3 |
|  |  |  | VIT_07s0129g00200 |  |  |  |  | -1,65 | 2,50E-02 | unknown | 3 |
|  |  |  | VIT_18s0001g02060 |  |  | -1,46 | 1,13E-04 | -2,00 | 1,26E-10 | Cyclin A1 | 3 |
|  |  |  | VIT_19s0085g00690 |  |  |  |  | -1,42 | 2,16E-04 | Cyclin-dependent protein kinase CYCB3 | 3 |
| Continue | | | | | | | | | | | |

**Supplementary Table 9.** (continued)

|  |  |  |  | **Aki** | | **BF** | | **BL** | |  | **Other** |
| --- | --- | --- | --- | --- | --- | --- | --- | --- | --- | --- | --- |
| **ID** | **GO ID** | **GO Description** | **Gene ID** | **log_2_(FC)** | **FDR** | **log_2_(FC)** | **FDR** | **log_2_(FC)** | **FDR** | **vCOST Description** | **ID*** |
| 7 | GO:0008284 | Positive regulation of cell population proliferation | VIT_00s0131g00080 | -1,79 | 1,36E-03 |  |  |  |  | Annexin ANN4 | 8 |
| 8 | GO:0009414 | Response to water deprivation | VIT_06s0004g04370 |  |  |  |  | -1,56 | 1,07E-17 | Histone H4 |  |
|  |  |  | VIT_13s0019g00780 |  |  |  |  | -1,87 | 6,94E-17 | Histone H4 |  |
|  |  |  | VIT_13s0019g00800 |  |  |  |  | -1,46 | 7,02E-05 | *Histone H4* |  |
|  |  |  | VIT_00s0131g00080 | -1,79 | 1,36E-03 |  |  |  |  | Annexin ANN4 | 7 |
| 9 | GO:0009909 | Regulation of flower development | VIT_14s0083g00640 |  |  | -1,82 | 4,54E-32 |  |  | Constans 2 (COL2) |  |
| 10 | GO:0010017 | Red or far-red light signaling pathway | VIT_04s0008g05210 |  |  | -1,56 | 2,64E-05 | -1,52 | 1,44E-02 | BZIP protein HY5 (HY5) |  |
|  |  |  | VIT_05s0020g01090 |  |  |  |  | -1,40 | 3,60E-13 | BZIP protein HY5 (HY5) |  |
| 11 | GO:0045910 | Negative regulation of DNA recombination | VIT_07s0005g01060 |  |  |  |  | -1,48 | 1,36E-05 | Histone H1 |  |
|  |  |  | VIT_07s0141g00730 |  |  |  |  | -1,41 | 2,14E-12 | Histone H1 |  |
|  |  |  | VIT_14s0081g00500 |  |  |  |  | -1,72 | 3,13E-17 | Histone H1 |  |
| 13 | GO:0009744 | Response to sucrose | VIT_01s0011g04400 |  |  |  |  | -1,63 | 3,85E-04 | Origin recognition complex subunit 4 |  |
|  |  |  | VIT_17s0000g01960 |  |  | -1,69 | 4,58E-02 | -1,92 | 7,87E-03 | *Origin recognition complex subunit 5* |  |
| 15 | GO:0010112 | Regulation of systemic acquired resistance | VIT_01s0011g03440 |  |  |  |  | -1,53 | 1,47E-09 | DNA mismatch repair protein |  |
| 16 | GO:0009627 | Systemic acquired resistance | VIT_00s0333g00050 |  |  |  |  | -1,58 | 1,09E-13 | DIR1 (defective IN induced resistance 1) |  |
|  |  |  | VIT_08s0007g01370 |  |  |  |  | -1,77 | 4,42E-03 | Protease inhibitor/seed storage/lipid transfer protein (LTP) |  |
| 17 | GO:0000076 | DNA replication checkpoint signaling | VIT_01s0010g01670 |  |  |  |  | -1,45 | 4,68E-03 | *Zinc knucle* |  |
|  |  |  | VIT_07s0104g01740 |  |  |  |  | -1,87 | 6,15E-04 | Protein kinase WEE1 |  |
| Continue | | | | | | | | | | | |

**Supplementary Table 9.** (continued)

|  |  |  |  | **Aki** | | **BF** | | **BL** | |  | **Other** |
| --- | --- | --- | --- | --- | --- | --- | --- | --- | --- | --- | --- |
| **ID** | **GO ID** | **GO Description** | **Gene ID** | **log_2_(FC)** | **FDR** | **log_2_(FC)** | **FDR** | **log_2_(FC)** | **FDR** | **vCOST Description** | **ID*** |
| 18 | GO:0045893 | Positive regulation of transcription | VIT_00s0541g00020 |  |  |  |  | -1,71 | 1,21E-03 | BZIP transcription factor BZIP6 |  |
|  |  |  | VIT_01s0026g01550 |  |  |  |  | -1,71 | 1,34E-08 | Homeodomain leucine zipper protein HB-1 |  |
|  |  |  | VIT_02s0025g02710 |  |  |  |  | -1,50 | 4,89E-03 | NAC Secondary wall thickening promoting factor1 |  |
|  |  |  | VIT_08s0007g04200 |  |  |  |  | -2,12 | 5,45E-05 | Late meristem identity1 HB51/LMI1 |  |
|  |  |  | VIT_08s0007g08150 |  |  |  |  | -1,45 | 5,00E-02 | *AP2/ERF domain-containing protein* |  |
|  |  |  | VIT_13s0067g02560 |  |  |  |  | -3,09 | 5,60E-21 | unknown |  |
|  |  |  | VIT_15s0048g02870 |  |  |  |  | -1,73 | 1,94E-07 | Homeobox-leucine zipper protein HB-7 |  |
| Akivi (Aki), lyophilized (BL) and fresh (BF) *Bacillus* UdG treatments | | | | | | | | | | | |
| ID: GO term assigned identifier. (*) the gene was also downregulated in this ID | | | | | | | | | | | |
| FDR, false discovery rate. Upregulated gene: log_2_(FC) ≥ -1.4 | | | | | | | | | | | |
| Genes in cursive are shared by ID 2, 3, 6 and 7 | | | | | | | | | | | |
| Description in cursive means that other databases were used instead of vCOST. | | | | | | | | | | | |

**Supplementary Table 10.** KEGG pathways influenced by Akivi (Aki), lyophilized (BL) and fresh (BF) *Bacillus* UdG treatments compared to the non-treated control (NTC).

| **Pathway ID** | **Pathway description** | **Number of DEGs** | **Corrected p-Value** |
| --- | --- | --- | --- |
| **Upregulated** |  |  |  |
| **Aki *vs* NTC** |  |  |  |
| vvi00480 | Glutathione metabolism | 23 | 2.05E-05 |
| vvi00591 | Linoleic acid metabolism | 7 | 1.95E-03 |
| vvi00592 | alpha-Linolenic acid metabolism | 11 | 8.75E-03 |
| vvi00900 | Terpenoid backbone biosynthesis | 10 | 9.82E-03 |
| vvi00410 | beta-Alanine metabolism | 10 | 9.82E-03 |
| vvi00071 | Fatty acid degradation | 8 | 4.35E-02 |
| **BL *vs* NTC** |  |  |  |
| vvi00500 | Starch and sucrose metabolism | 32 | 9.80E-04 |
| **BF *vs* NTC** |  |  |  |
| vvi00500 | Starch and sucrose metabolism | 25 | 2.37E-02 |
| vvi03008 | Ribosome biogenesis in eukaryotes | 17 | 2.44E-02 |
|  |  |  |  |
| **Downregulated** |  |  |  |
| **Aki *vs* NTC** |  |  |  |
| vvi00860 | Porphyrin and chlorophyll metabolism | 11 | 2.37E-03 |
| **BL *vs* NTC** |  |  |  |
| vvi03030 | DNA replication | 17 | 2.60E-04 |
| vvi00270 | Cysteine and methionine metabolism | 21 | 2.06E-02 |
| **BF *vs* NTC** |  |  |  |
| vvi00051 | Fructose and mannose metabolism | 12 | 2.56E-02 |
| vvi00940 | Phenylpropanoid biosynthesis | 24 | 3.10E-02 |
| vvi03030 | DNA replication | 10 | 3.10E-02 |
| vvi00500 | Starch and sucrose metabolism | 19 | 3.64E-02 |

**Supplementary Table 11.** Mineral nutrient concentrations in leaves of grapevine cvs. Garnacha Blanca, Garnacha Tinta, and Macabeo treated with Akivi (Aki), lyophilized *Bacillus* UdG (BL), or water (NTC). Data show mean values ± standard deviation of three biological replicates. Significant differences according to the Tukey test (parametric tests) or the Dunn test (non-parametric tests) between treatment (Aki or BL) and NTC are represented by asterisks (*).

|  | **‘Garnacha Blanca’** | | | | | | **'Garnacha Tinta’** | | | | | | **‘Macabeo’** | | | | | |
| --- | --- | --- | --- | --- | --- | --- | --- | --- | --- | --- | --- | --- | --- | --- | --- | --- | --- | --- |
| **Codes** | **NTC** | | **Aki** | | **BL** | | **NTC** | | **Aki** | | **BL** | | **NTC** | | **Aki** | | **BL** | |
| **Macronutrient (mg/g DW)** | | | | | | | | | | | | | | | | | | |
| **P** | 7.02 | ±0.16 | 7.06 | ±0.03 | 7.77 | ±0.12 | 7.64 | ±0.51 | 9.91 | ±0.13 | 8.34 | ±0.1 | 5.81 | ±1.03 | 6.19 | ±0.56 | 6.84 | ±0.21 |
| **K** | 18.94 | ±0.34 | 19.34 | ±1,00 | 18.17 | ±0.63 | 19.22 | ±0.82 | 23.32 | ±1.57 | 17.31 | ±0.69 | 15.18 | ±3.51 | 16.46 | ±1.45 | 16.28 | ±0.79 |
| **Ca** | 17.94 | ±0.44 | 17.99 | ±0.26 | 17.88 | ±0.49 | 13.49 | ±1.1 | 11.05 | ±3.05 | 15.73 | ±0.17 | 14.73 | ±1.33 | 17.95 | ±0.82 | 16.94 | ±0.66 |
| **Mg** | 3.23 | ±0.18 | 3.26 | ±0.05 | 3.10 | ±0.1 | 2.74 | ±0.15 | 3.07 | ±0.06 | 2.98 | ±0.11 | 2.55 | ±0.35 | 2.98 | ±0.2 | 2.91 | ±0.18 |
| **S** | 3.62 | ±0.15 | 3.58 | ±0.02 | 3.52 | ±0.03 | 3.10 | ±0.24 | 3.71 | ±0.04 | 3.45 | ±0.04 | 3.42 | ±0.49 | 3.48 | ±0.03 | 3.61 | ±0.08 |
| **Micronutrient (µg/g DW)** | | | | | | | | | | | | | | | | | | |
| **Fe** | 154.3 | ±9.3 | 153.3 | ±10.5 | 155.5 | ±5.9 | 104.1 | ±12.9 | 123.8 | ±6.4 | 129.2 | ±2.8 | 300.7 | ±31,0 | 194.3* | ±22.7 | 164.9* | ±24.6 |
| **Mn** | 124.7 | ±9.7 | 122.0 | ±7.2 | 122.0 | ±2.2 | 187.1 | ±20.5 | 184.6 | ±20.6 | 208.3 | ±12.2 | 95.1 | ±10.5 | 112.0 | ±5.3 | 120.0 | ±8.2 |
| **Na** | 171.4 | ±23.5 | 91.0 | ±25,0 | 211.2 | ±17.4 | 131.6 | ±12.9 | 161.0 | ±45.2 | 275.9 | ±9.6 | 375.2 | ±29.4 | 358.6 | ±35.9 | 616.7 | ±75.6 |
| **B** | 37.8 | ±1.2 | 34.5 | ±0.9 | 35.6 | ±0.3 | 35.0 | ±3.3 | 39.7 | ±1.5 | 34.2 | ±0.1 | 27.5 | ±6.3 | 36.1 | ±0.3 | 31.1 | ±1.7 |
| **Cu** | 16.2 | ±1.8 | 13.1 | ±1.4 | 10.7* | ±0.5 | 6.7 | ±1.2 | 8.1 | ±2.2 | 8.4 | ±0.2 | n.d. |  | n.d. |  | 11.9 | ±1.6 |
| **Zn** | 35.5 | ±3.3 | 30.2 | ±1.8 | 28.3 | ±0.3 | 29.1 | ±1.9 | 31.1 | ±2.7 | 32.7 | ±1.7 | 30.2 | ±3.4 | 34.9 | ±3.1 | 36.9 | ±1,0 |
| Macronutrient: P, phosphorus ; K, potassium ; Ca, calcium ; Mg, magnesium; S: sulphur | | | | | | | | | | | | | | | | | | |
| Micronutrient: Mn, manganese; Cu, copper; B, boron; Fe, iron; Na, sodium; Zn, zinc | | | | | | | | | | | | | | | | | | |
| n.d. no determined | | | | | | | | | | | | | | | | | | |
| Mineral nutrient analysis: The mineral nutrients were extracted with a classical extraction protocol. Briefly, 100 mg of fresh grapevine leave powder was dried and acid pre-digested overnight with 7 mL of solvent (HNO_3_:H_2_O_2_ 69 %: 30 %, 5:2 v/v) and then digested in a hot-block digestion system (SC154-54-Well Hot Block™, Environmental Express, SC, USA) at 110 °C for 4 h. The digested samples were adjusted at 25mL with H_2_O milli-Q and filtrated at 45µm prior to nutrient element concentrations measurements by ICP-OES (Perkin Elmer Optima 8300, MA, USA). Blanks were included in each batch of samples for quality control. | | | | | | | | | | | | | | | | | | |

**
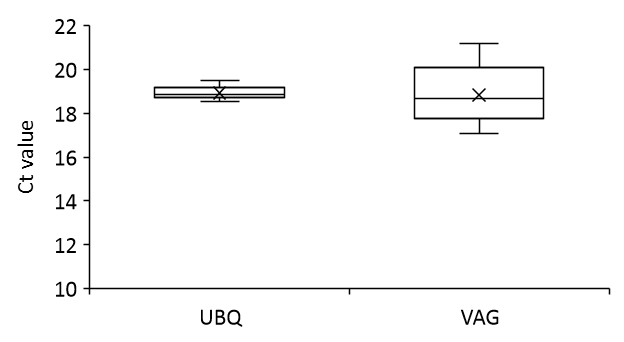
**

**Supplementary Figure 1.** Evaluation and validation of the most appropriate endogenous gene to normalize gene expression data according to the method described by Silver et al., (2006). VAG gene coding for the Vacuolar ATPase subunit G (GenBank accession number XM_002281110.1) and UBQ gene coding for the Ubiquitin-conjugating enzyme (GenBank accession number EC922622) (Monteiro et al., 2013). Expression level stability of endogenous gene candidates between the four treatments (non-treated control, Akivi, lyophilized and fresh *Bacillus* UdG). Boxplots comparing Ct values of UBQ and VAG between treatments.

**Supplementary Figure 2.** Principal component analysis (PCA) of the biological replicates of Akivi (A and B), lyophilized (C and D), and fresh (E and F) *Bacillus* UdG treatments (white symbol) compared to the non-treated control (black symbol). In the left panels (A, C, and E) the four biological replicates (R1, R2, R3 and R4) are shown in the PCA for each modality. The three selected replicates for further analysis are represented by circles and the odd replicates are represented by squares. The right panels (B, D, and F) show the PCA of the filtered three replicates presenting less variability for each modality.

**
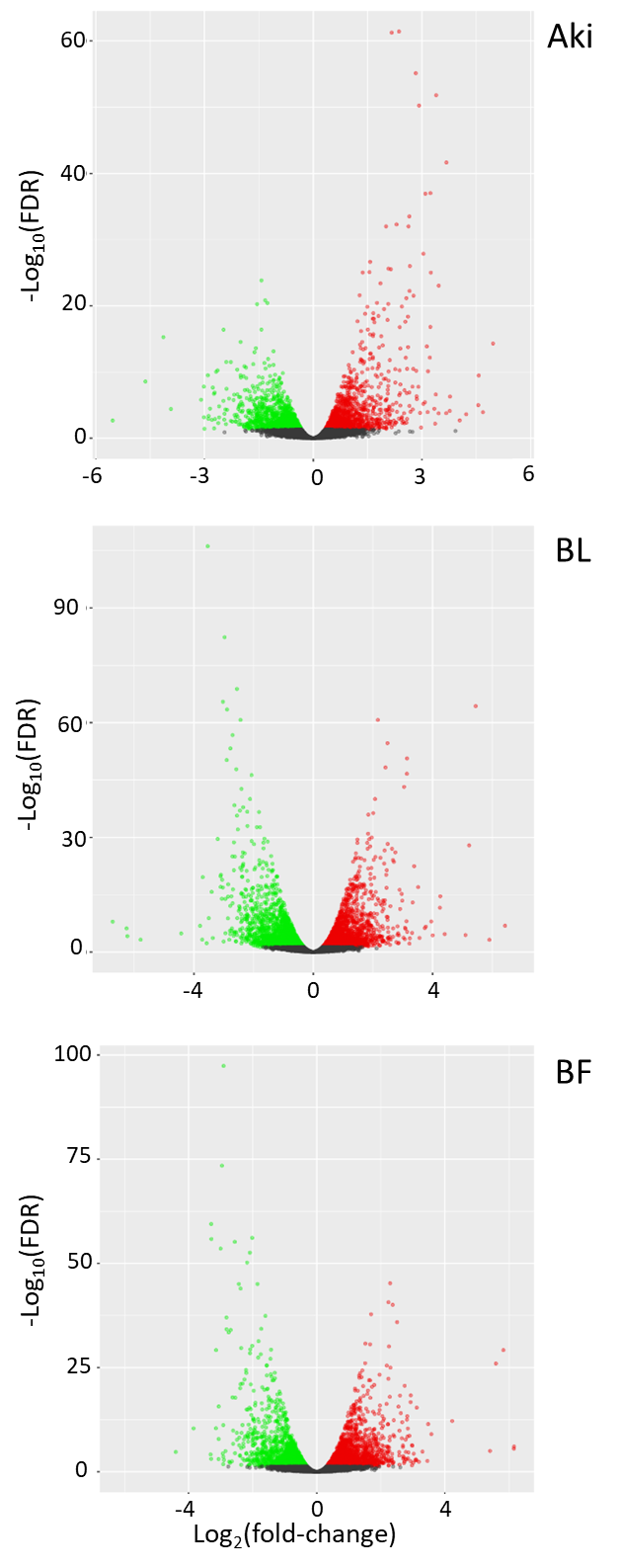
**

**Supplementary Figure 3.** Volcano plots of differentially expressed genes (DEGs) in grapevine leaves comparing the treatments Akivi (Aki), lyophilized (BL) and fresh (BF) *Bacillus* UdG to the non-treated control (NTC). FDR: False discovery rate (adjusted P-values). Black dots represent the genes that are not significantly differentially expressed, while red and green dots are the gens that are significantly up- and downregulated, respectively. The most upregulated genes are towards the right, the most downregulated genes are towards the left, and the most statistically significant genes are towards the top.

**
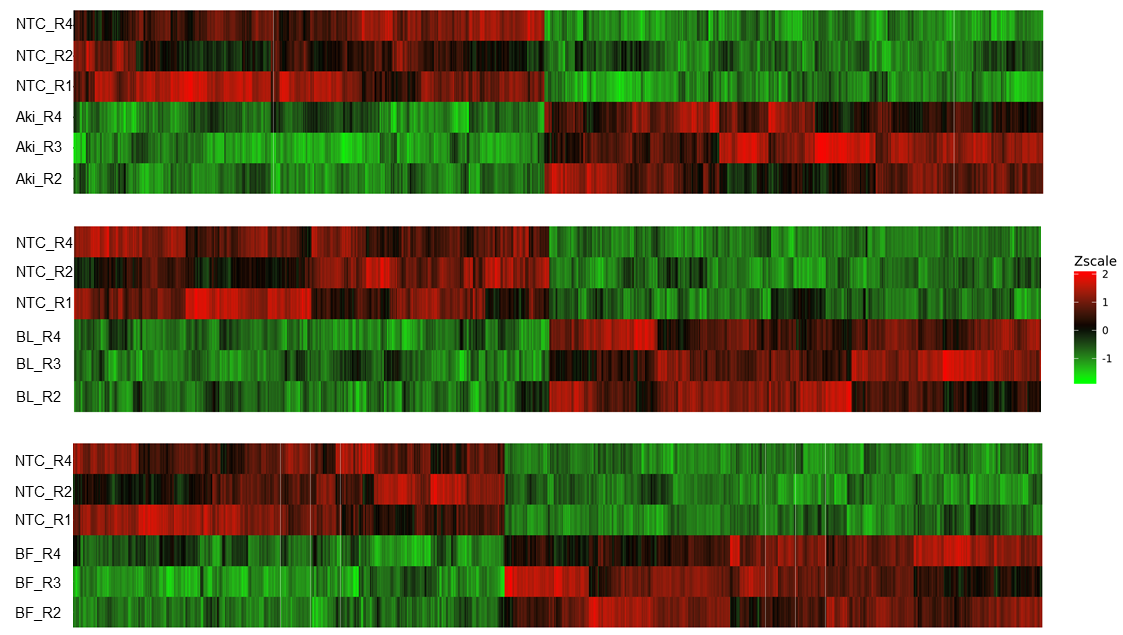
**

**Supplementary Figure 4**. Heatmaps of the differentially expressed genes (DEGs) (Z-scaled FPKM values) showing the results from the different treatments (Akivi, Aki; lyophilized, BL, and fresh, BF, *Bacillus* UdG) compared to the non-treated control (NTC). Changes in expression levels are displayed from green (down-expressed) to red (over-expressed). The order of the genes was established after hierarchical clustering using the Euclidean distance X.


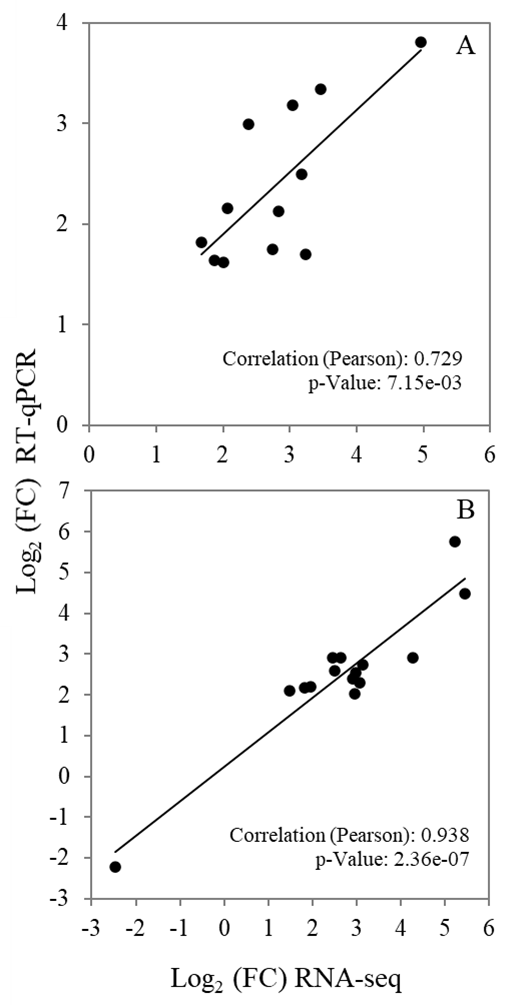


**Supplementary Figure 5.** Relationship between RNA-seq and RT-qPCR transcriptomic data of selected genes in leaves of cv. Garnacha Blanca grapevine after treatment with Akivi (A) and lyophilized *Bacillus* UdG (B), respectively. Data shown as Log_2_ (FC), where FC means fold-change. The Pearson’s correlation coefficient between relative expression levels is also shown.
